# Fe- and Ru-H-Mordenites for Polyethylene Upcycling: Insights from Thermo-Catalytic Pyrolysis and DFT Studies

**DOI:** 10.1021/acssuschemeng.5c11349

**Published:** 2026-03-30

**Authors:** Gita Pandey, Sujoy Bepari, Sumit Gupta, Tianjun Xie, Debasish Kuila

**Affiliations:** † Department of Chemistry, 3616 North Carolina Agricultural and Technical State University, Greensboro, North Carolina 27411, United States; ‡ Joint School of Nanoscience and Nanoengineering, North Carolina Agricultural and Technical State University, Greensboro, North Carolina 27411, United States; § Department of Chemical, Biological and Bioengineering, North Carolina Agricultural and Technical State University, Greensboro, North Carolina 27411, United States

**Keywords:** thermo-catalytic pyrolysis, polyethylene (PE), H-mordenite (HM), propene, H_2_

## Abstract

Catalytic pyrolysis of polyethylene (PE) was performed using Fe- and Ru-impregnated H-mordenite (HM) catalysts to produce lighter hydrocarbons. Catalyst surface areas were analyzed by N_2_ adsorption−desorption and acidity by NH_3_-TPD studies. Reducibility of metal from H_2_-TPR, experiments indicate multiple oxidation states of Fe due to higher H_2_ consumption. The catalyst activity studies monitored by GC–MS analysis highlight the importance of temperature and PE: catalyst ratio in optimizing hydrocarbon selectivity to C_1_–C_4_ alkanes and C_2_–C_5_ olefins. Studies with polymer-to-catalyst ratios of 1:1 and 1:2 at higher temperature increased PE conversion with stronger acid sites favoring conversion and moderate acid sites improving selectivity toward lighter olefins. While propane dominated at lower temperatures, propene was the main product at 500 °C. Both catalysts exhibited overall similar conversion, while Ru-HM yielded >40% propene selectivity compared to <40% for Fe-HM. Ethylene selectivity exceeded 10% in both cases. Density functional theory simulations using a C_4_ surrogate on Ru-HM confirmed that late-stage cracking dominates dehydrogenation, validating the proposed reaction mechanism.

## Graphical abstract

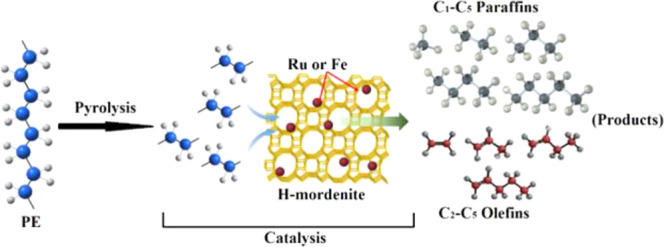

## 1 Introduction

Plastics, synthetic or semisynthetic materials primarily composed of long-chain polymers, have become indispensable in modern society. Global production has now exceeded 400 million tons annually, accompanied by a steady rise in solid waste generation over the past five decades.^1^ Single-use plastics, designed for short-term applications, are particularly problematic due to their low recyclability and persistence in the environment. Their nonbiodegradable nature leads to long-term accumulation in ecosystems, causing ecological imbalance and health risks to wildlife.^2^ Although several recycling methods exist, landfill disposal remains the dominant practice in many developing countries.^2^ Landfilling not only creates public health hazards through insect and rodent proliferation but also incurs substantial economic losses, with an estimated $7.2 billion of potential value wasted annually in the United States.^3^

Among plastics, polyethylene (PE), the most widely consumed plastic, accounts for nearly 48% of the total demand.^4^ With annual production exceeding 40 million tons for packaging, construction, automotive, medical, agricultural, and electronic applications, PE has become a major contributor to plastic pollution. Its durability, while valuable for performance, makes it especially challenging for degradation or recycling. Converting PE into value-added products therefore presents a challenge to mitigate environmental impacts while simultaneously promoting a circular economy.^5^

Thermochemical conversion, such as pyrolysis, has emerged as an attractive strategy for valorizing plastic waste. PE possesses a high calorific value, making it suitable for conversion into fuels and chemicals that can offset fossil fuel consumption.^6^ In pyrolysis, plastics are typically heated between 400 and 500 °C under oxygen-free conditions, producing gaseous, liquid, and solid fractions.^7^ This process, rooted in petrochemical technologies, allows recovery of hydrocarbons embedded within polymers and thereby addresses two pressing challenges: plastic waste management and energy resource diversification.^8^ However, thermal pyrolysis alone often results in low selectivity and requires high energy input due to the complex hydrocarbon structures of polyolefins such as high-density polyethylene (HDPE), low-density polyethylene (LDPE), and polypropylene (PP).^9,10^ Catalysts are therefore essential to improving efficiency, lowering energy demand, and tailoring product distribution. Zeolites, silica−alumina, and fluid catalytic cracking catalysts have been widely investigated for this purpose.^11,12^ Among these, zeolites—particularly HZSM-5, H-mordenite (HM), and HY—are especially promising due to their strong acidity, shape-selectivity, and ability to crack long polymer chains into shorter, fuel-like hydrocarbons.^4,13^ For example, HM, a large-pore zeolite, enables efficient cracking of PE into gasoline-range molecules by acting as a molecular sieve.^4,13^ Recent studies have further demonstrated that hierarchical zeolites and metal-modified variants can significantly enhance stability, improve hydrocarbon selectivity, and increase aromatic yields.^14–16^ These findings underscore the potential of zeolite-based catalysts for advancing plastic-to-fuel technologies.

The present study investigates thermo-catalytic pyrolysis of polyethylene using H-mordenite (HM) and its transition-metal-incorporated derivatives. Iron (Fe) and ruthenium (Ru) were added to HM to enhance the catalytic activity and selectivity toward light olefins and aromatic products. Special attention was given to the role of the Si/Al ratio in determining acidity and stability and how metal incorporation modulates active site properties. Furthermore, mechanistic insights into the pyrolysis of PE on Ru-HM were explored using first-principles calculations, allowing the proposal of competitive reaction pathways. Experimental reaction rates are compared with theoretical predictions, offering a comprehensive understanding of the catalytic mechanisms underlying PE conversion. Despite extensive research on the catalytic pyrolysis of polyethylene, several key challenges remain in controlling product selectivity toward light olefins and in establishing mechanistic links among catalyst structure, temperature, and cracking pathways. Many prior studies report product distributions without quantitatively rationalizing the role of metal−acid bifunctionality or providing kinetic context for temperature-dependent trends.

In this work, we address these gaps by systematically investigating thermo-catalytic PE pyrolysis over H-mordenite modified with Fe and Ru metals under reduced conditions. The innovation of this study lies in correlating catalyst acidity, metal dispersion, and the effect of temperature on experimentally measured apparent reaction rates and product selectivity, supported by density functional theory (DFT) calculations using a C4 surrogate model. By integration of experimental observations with theoretical energy-span analysis, this work provides mechanistic insight into dehydrogenation, cracking, and reaction pathways. The results establish structure–performance relationships that are directly relevant to designing catalysts for selective plastic upcycling.

## 2 Materials and Methods

### 2.1 Materials

The PE pellets (5 MM in size; average molecular weight (376,270 g mol^−1^)) were obtained from DOW chemicals, USA. The commercial H-mordenite (HM) zeolite was procured from ACS Material, LLC, USA. The metal precursors such as iron nitrate nonahydrate [Fe­(NO_3_)_2_·9H_2_O] and ruthenium chloride [RuCl_3_·*x*H_2_O] were purchased from Alfa Aesar, USA. Ethanol, used as a solvent, was acquired from Fischer Scientific, USA.

### 2.2 Catalyst Synthesis

The metal-impregnated catalysts were synthesized by an incipient wet-impregnation method. The stoichiometric molar ratios of iron nitrate and ruthenium chloride were mixed separately according to the desired metal loading (5 wt %) and dissolved in ethanol to prepare the aqueous solution. The volume of the solution used for each catalyst preparation was determined by the total pore volume of the catalyst support (HM). The total pore volume of HM was obtained from the BET analysis. The aqueous solution of each metal (Fe, Ru) salt was mixed thoroughly with HM support and stirred vigorously (500 rpm) at 40 °C for 1 h. The whole solution was aged for 24 h at room temperature (25 °C). The solution was dried in an oven at 90 °C. Finally, the two catalysts were calcined at 550 °C for 6 h in a furnace. The prepared catalysts were designated as Fe-HM and Ru-HM.

### 2.3 Catalysts Characterization

Characterization of the catalysts included multiple analytical techniques. X-ray diffraction (XRD, Bruker AXS) using Cu Kα_1_ radiation (λ = 1.5406 Å) was performed over 2θ = 5−80°. Fourier Transform Infrared Spectroscopy (FTIR, Shimadzu IR Prestige-21) identified functional groups within 400−4000 cm^−1^. X-ray photoelectron spectroscopy (XPS, Thermo-Scientific Escalab Xi+) analyzed the surface chemical states of Al and Si. Surface area and porosity were measured via N_2_ adsorption−desorption (BET, Micromeritics 3-Flex ASAP 2020) with BJH analysis after 6 h degassing at 120 °C. Morphology and particle size were examined by FESEM (JEOL JSM-IT800) and TEM (Thermo-Fisher Talos F200X, 200 kV). Temperature-programmed reduction (H_2_-TPR, Micromeritics 3-Flex) was conducted on 0.05 g samples in 10% H_2_/Ar from room temperature to 1000 °C at 10 °C/min. Acidity was measured via a NH_3_-TPD (Micromeritics Autochem 2920). Thermal stability was evaluated by TGA-DSC (TA Instruments) under 100 mL/min air flow, heating to 1000 °C at 10 °C/min.

### 2.4 Thermo-Catalytic Pyrolysis of Polyethylene

Thermo-catalytic pyrolysis of PE was carried out in a fixed-bed reactor (0.25 in. inner diameter and 14.5 in. length). The two-stage bed consisting of PE and catalysts was used inside the reactor for the pyrolysis process. Nitrogen (N_2_) was used as an inert carrier gas in the experimental setup to keep the pyrolysis environment oxygen-free. The N_2_ flow rate was adjusted to 1.5 mL/min during pyrolysis. Figure 1 shows the schematic diagram of the pyrolysis setup.^13^ The catalytic pyrolysis experiments were conducted using carefully prepared mixtures of PE and catalyst samples in weight ratios of 1:1 and 1:2. These mixtures were subjected to a controlled heating rate of 10 °C/min, with target temperatures set at 400, 450, and 500 °C. To reach the desired reaction temperature, the reactor was enclosed in a vertical split-tube furnace within the experimental apparatus, which provided the necessary thermal energy. As pyrolysis progressed, condensable liquid products were collected via a condenser system. The noncondensable gaseous products were directly passed through online GC–MS (Shimadzu GCMS-QP2020NX) for quantification. After each experiment, the solid residue remaining in the reactor was accurately weighed. This systematic approach demonstrated the efficacy of the catalytic pyrolysis method in converting PE to valuable products, providing a comprehensive analysis of the product distribution across different temperature ranges and catalyst ratios. The liquid and gaseous products were analyzed using a GC–MS instrument equipped with a Restek RTX-1 MS capillary column. Helium served as the carrier gas with a split ratio of 400:1. The temperature program began at 40 °C for 2 min, then increased from 35 to 100 °C at a rate of 20 °C/min for 1 min, followed by an increase from 100 to 150 °C at 30 °C/min for another minute. The mass range for MS detection was set from 16 to 250 amu (*m*/*z*), with the ion source temperature established at 200 °C. The reaction temperature was selected based on TGA analysis shown in Figures S3−S5.

**Figure 1:**
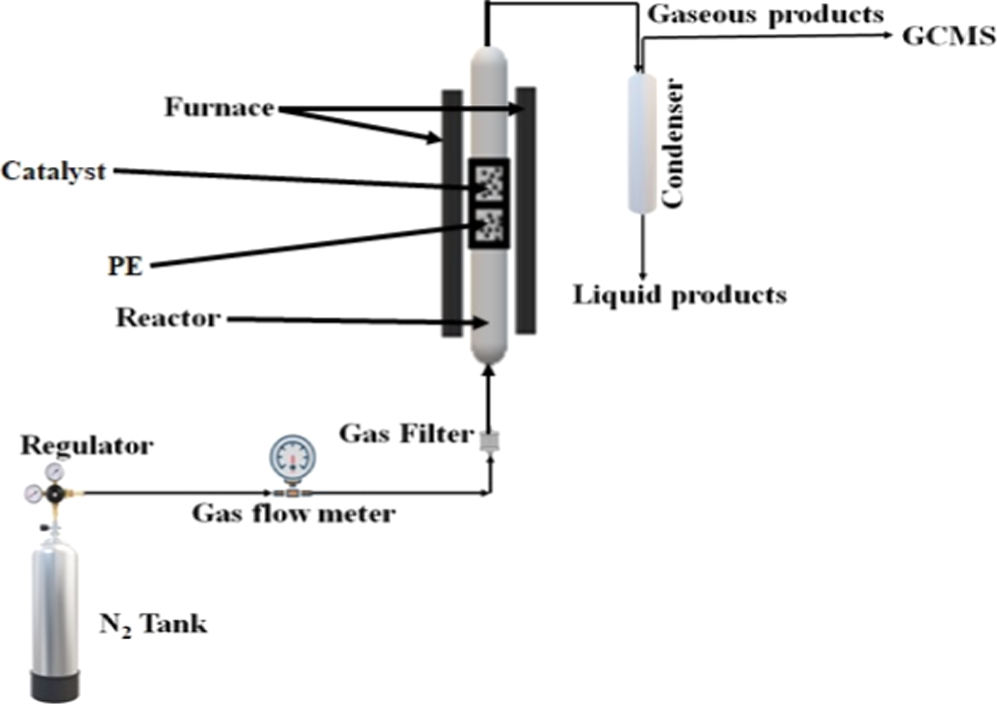
Schematic diagram of pyrolysis setup.

The PE conversion and selectivity of product *i* (*S*_*i*_) were calculated using the following equations.

1 $$PE\;conversion\;\left(\%\right)=\left(\frac{p_{i}-p_{o}}{p_{i}}\right)\times 100$$

where *p*_i_ and *p*_o_ are the initial and final weight of PE.

2 $$S_{i}\;\left(\%\right)=\frac{\left[n_{i}\right]}{\sum n_{gas}}\times 100$$

where [*n*_*i*_] is the number of moles of carbon in product *i*, and *n*_gas_ is the total number of moles of carbon in all quantified gaseous products.

## 3 Results and Discussion

### 3.1 Characterizations of Catalysts

#### 3.1.1 N_2_ Adsorption−Desorption Isotherm (BET)

The textural properties of the catalysts are shown in Figure 2. According to Figure 2a, all the samples depicted type IV isotherms with a H4-type hysteresis loop, indicating the hierarchical porous texture.^17^ The presence of mesopores is confirmed by the hysteresis loop from 0.45 to 0.95 P/P_0_.^17^ The hysteresis loop is observed at the high-pressure range, indicating mostly the presence of mesoporous range from the stacking spaces of crystal.^18^ The pore size distribution plot (Figure 2b) shows that the highest number of pores falls within the mesopore range (2 to 50 nm).

**Figure 2:**
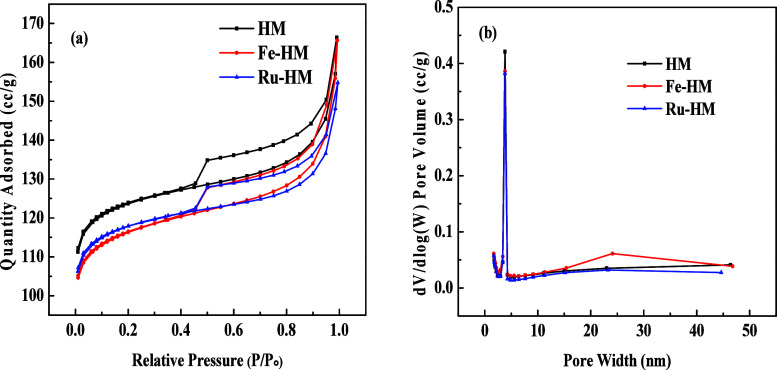
N_2_ adsorption−desorption isotherms (BET) of all catalysts: (a) isotherm plot; (b) pore size distribution.

The physicochemical properties of the catalysts are listed in Table 1. H-Mordenite (HM) zeolite with topology (pore opening with a 12-membered ring, approximately 0.7 nm) is used as support to investigate the effect of pore structures.^19^ The specific surface area of the parent support (HM) is 374.73 m^2^/g. After incorporation of Fe and Ru metal oxides, the surface area of HM decreases. This result reflects that some pores are partially blocked by Fe and Ru metals, which causes a decrease in the surface area. The Fe-HM and Ru-HM catalyst’s surface areas are 354.42 and 357.47 m^2^/g, respectively. The pore volume is not affected that much upon incorporation of metal oxides. The small increase in the pore width for Fe-HM can be attributed to a minor etching effect during the impregnation process, which could expand some pores to some extent. In contrast, the decrease in the pore diameter for Ru-HM shows that Ru particles are partially clogging some pores, effectively reducing their average diameter.

**Table 1:** Physicochemical Properties of All Catalysts

catalyst	surface area (m^2^/g)	pore volume (cc/g)	pore diameter (nm)
HM	374.73	0.26	2.75
Fe-HM	354.42	0.26	2.88
Ru-HM	357.47	0.24	2.66

#### 3.1.2 FTIR Analysis

The vibrational bands in the catalysts were investigated by FTIR spectroscopy. The FTIR absorbance bands are listed in Figure 3. The absorption bands of all catalysts were observed at 1631, 1223, 1055, 800, 567, and 433 cm^−1^.^20^ The peak intensity decreased with the incorporation of Fe and Ru in the zeolite. The diminished intensity suggests that Fe and Ru metals are absorbed on the surface. The external and internal symmetric and asymmetric stretching vibrations and T–O bending vibration are the reasons for the peaks in absorption spectrum at 433, 567, 800, 1055, and 1223 cm^−1^.^17^ The absorption of IR light by water causes the water molecules within the zeolite structure to bend and vibrate. The water molecule’s bending vibration is observed at 1631 cm^−1^.^17^ The presence of the Si–O–Si bond is observed as a broad peak at 1055 cm^−1^ and a bending vibration at 800 cm^−1^. The SiO_4_ and AlO_4_ tetrahedral units of double five-ring (D5R) units are observed at 567 cm^−1^.^21,22^ These spectral features indicate that the chemical properties of the zeolite framework remain largely unchanged during the formation of hierarchical pores, suggesting that the integrity of the catalyst’s structure is maintained throughout the modification process or after incorporation of metals such as Fe and Ru. This is significant, as it implies that the fundamental catalytic properties of zeolite are likely to be retained; in other words, the active sites within the zeolite framework remain unchanged.

**Figure 3:**
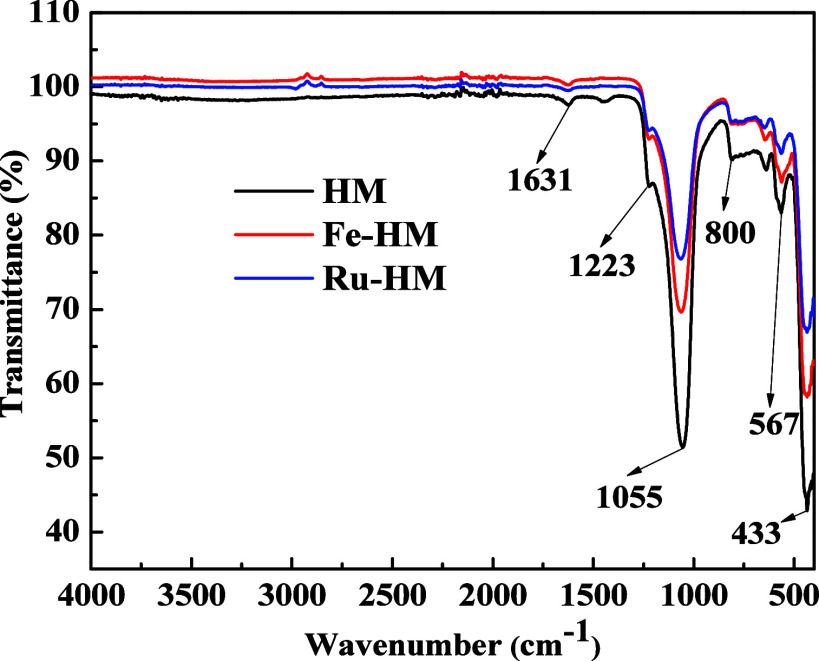
FTIR analyses of all catalysts prepared as such (As).

#### 3.1.3 FESEM Analysis

The morphologies of the HM, Fe-HM, and Ru-HM samples were examined by FESEM studies. The FESEM images show that the HM zeolite structure resembled the pillar-assembled structure (Figure 4a),^23^ and the shape of the particles remained unaffected after incorporation of Fe and Ru. Some small particles are well distributed over the support of the HM (Figure 4b,c), which could be due to Fe and Ru particles. The addition of Fe and Ru particles resulted in more homogeneous smaller particles.

**Figure 4:**
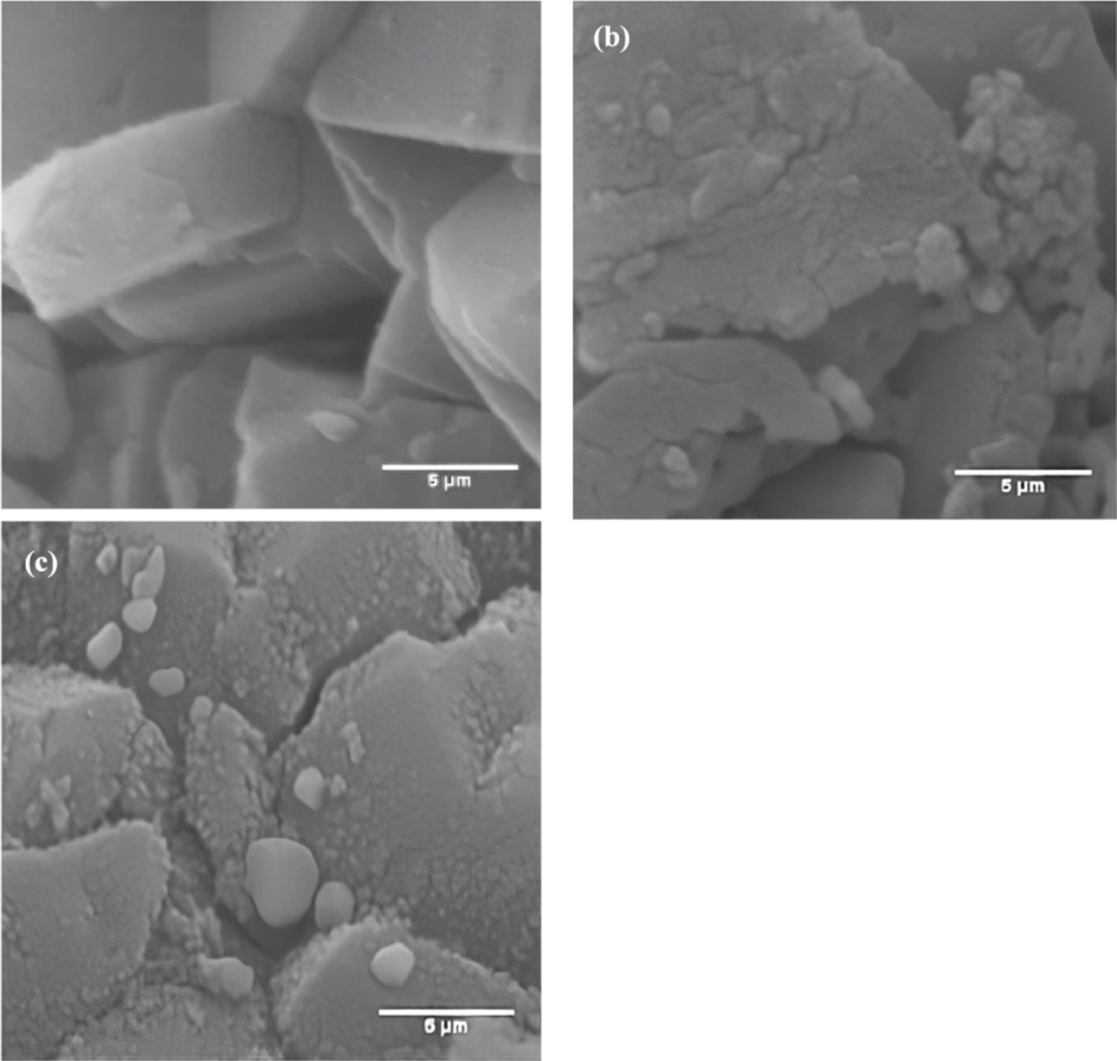
FESEM images of: (a) HM; (b) Fe-HM; (c) Ru-HM samples.

The SEM-EDS analysis was conducted for Fe-HM and Ru-HM catalysts. The EDS images are presented in Figure S1 and Figure S2, respectively. The figures delineate a uniform distribution of metals across the zeolite surface. The measured concentrations of O, Al, and Si in two catalyst samples exhibit minimal variation, suggesting that the fundamental properties of the HM zeolite remain the same after metal incorporation (Table 2). These results suggest the stability of the zeolite structure after the addition of metals. In the case of the Fe-HM catalyst, the metal loading of Fe (5.4 wt %) is slightly higher than intended loading (5 wt %), indicating that Fe is more concentrated on the HM zeolite surface. This means that Fe is deposited on the external surface of the zeolite particles rather than diffusing into the internal pores. Hence, higher concentrations of Fe could increase the accessibility of active sites during thermo-catalytic pyrolysis. For the Ru-HM catalyst, the measured loading of Ru (2.7 wt %) is less than the intended loading (5 wt %), suggesting that Ru may be distributed within the pore of the zeolite, with less distribution on the zeolite surface.^24,25^ The presence of Ru within the zeolite pores could increase specific catalytic properties due to the unique confinement in the framework of zeolites.

**Table 2:** SEM-EDS Results of Fe-HM and Ru-HM Catalysts

	loading (wt %)				
catalyst	Si	Al	Fe	Ru	O
Fe-HM	42.2	3.6	5.4		48.8
Ru-HM	42.6	3.4		2.7	51.3

#### 3.1.4 X-ray Photoelectron Microscopy

X-ray photoelectron spectroscopy was performed to analyze the chemical composition and coordination between metal and H-mordenite. The presence of O, Si, Al, Ru, and Fe is confirmed from the survey spectrum of HM and Metal (Ru and Fe)-incorporated HM (Figure 5). A Shirley background was applied to account for inelastic electron scattering, which is appropriate for transition metal oxides. XRD also (Figure 8b,d) confirms the presence of Fe_2_O_3_ in the bulk catalyst. As Fe exhibits mixed oxidation states with Fe^3+^ as the dominant species, the XPS reveals a mixed Fe^2+^/Fe^3+^ surface composition, indicating partial surface reduction.^26^ In Figure 6a, the XPS elemental analysis of Fe-HM shows binding energies near 710.6 and 724.1 eV, corresponding to Fe^3+^ 2p_3/2_ and Fe^3+^ 2p_1/2_, respectively, which confirm the presence of Fe^3+^ species.^27^ The coexistence of multiple oxidation states probably contributes to the redox cycling during PE pyrolysis. The satellite peaks (719 and 732.3 eV) are highly sensitive to the oxidation states of Fe and aids in the qualitative identification of the iron oxidation state.^28^ The shift in binding energy (BE) may be due to the presence of a higher electronegative silicon ion.

**Figure 5:**
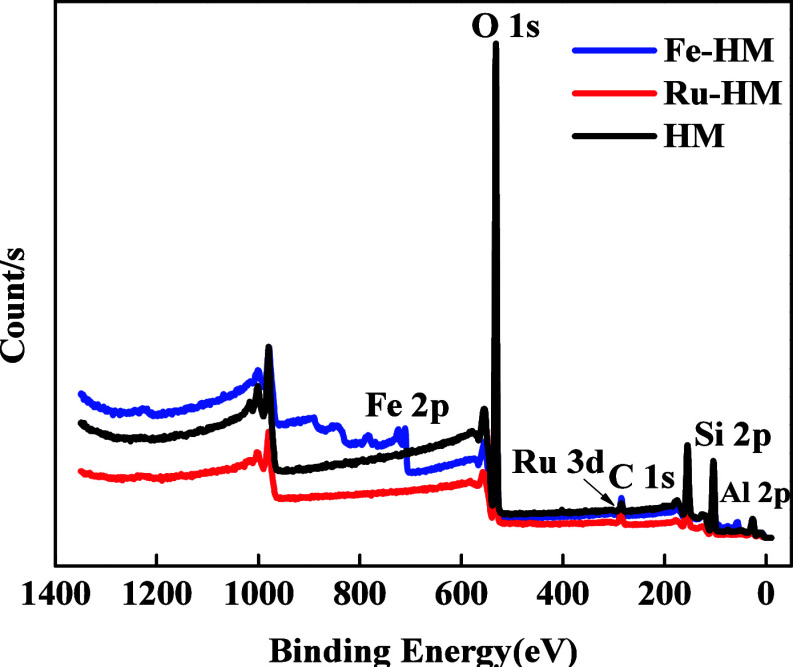
XPS survey of Fe-HM, Ru-HM, and HM Catalysts.

**Figure 6:**
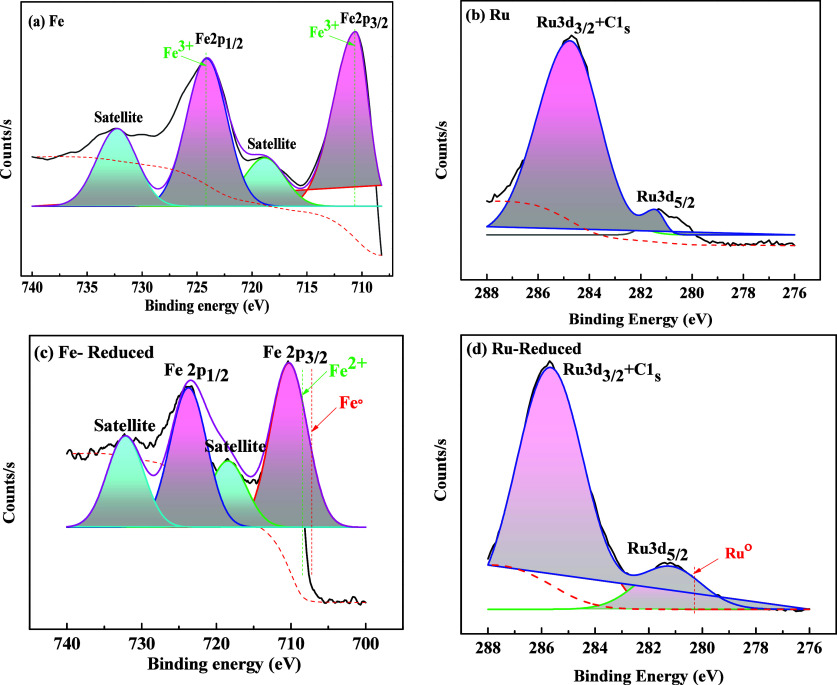
XPS elemental analyses of: (a) Fe; (b) Ru; (c) Fe-Reduced; and (d) Ru-Reduced Modernite Catalysts.

A Shirley background was applied for the Ru-HM catalyst. In Figure 6b, the BE within 278 to 288 eV indicates the presence of Ru 3d. A prominent peak at 284.2 eV was observed due to the strong C 1s signal of carbon.^29^ In Ru-HM, no oxidation state of Ru is observed; two different binding energies at 281.4 and 286 eV represent the oxidation state of Ru 3d_5/2_ and Ru 3d_3/2_.^30,31^ The XPS profiles of reduced Fe and Ru samples are shown in Figure 6c,d, respectively. After reduction at 550 °C in an Ar +10% H_2_ (v/v) atmosphere, two shoulders at lower BE indicate reduction of Fe^3+^ to Fe^2+^ (BE ∼ 710.3 and 723.6 eV for Fe^2+^ 2p_3/2_ and Fe^2+^ 2p_1/2_ (green dotted line)) and metallic Fe° (BE ∼ 707.1 eV (red dotted line)).^26,32^ For Fe^2+^ satellites, the binding energies of 718.5 and 732.1 eV are considered.^33^ In the case of the reduced Ru sample, metallic Ru° is observed at BE ∼ 280.2 eV.^34^ The Ru 3d_5/2_ oxidation state is observed at a BE of 281.2 eV. The Ru 3d_3/2_ and C 1s are superimposed at a BE of 285.6 eV. As discussed before, Ru^4+^ is dominant prior to reduction, while metallic Ru° emerges after reduction, providing active sites for hydrogenation–dehydrogenation reactions. These results, consistent with TPR analysis, suggest that Fe offers stronger redox cycling capacity, whereas Ru exhibits easier reducibility, favoring hydrogen transfer and propene selectivity as discussed in Section 3.1.7.

#### 3.1.5 TEM Analysis

The microstructures of the HM, Fe-HM, and Ru-HM catalysts were investigated by TEM studies (Figure 7). The representative TEM image reveals smooth lattice fringes, highlighting the well-defined crystalline structure of the parent HM (Figure 7a).^35^ The images confirm that the crystalline ordering of HM is preserved after metal impregnation. Fe_2_O_3_ (∼18.8 nm) and RuO_2_ (∼24.4 nm) crystallites are uniformly distributed across the zeolite framework without significant agglomeration (Figure 7b,c). The presence of both micro- and mesopores is evident, which facilitates the diffusion of PE-derived intermediates and supports secondary cracking during pyrolysis. High-resolution TEM shows lattice fringes corresponding to the HM framework, demonstrating the retention of the structural integrity of the zeolite after Fe and Ru incorporation. These results are consistent with our XRD analyses described below. While conventional TEM images do not directly resolve micro- or mesoporous features, the presence of hierarchical porosity is evidenced by N_2_ adsorption−desorption isotherms and pore size distribution analyses (Figure 2), which reveal both microporous and mesoporous contributions. TEM primarily confirms the preservation of crystalline ordering and the uniform dispersion of Fe_2_O_3_ and RuO_2_ nanoparticles. TEM-EDS elemental mapping confirms the presence and homogeneous nanoscale distribution of Fe and Ru species on H-mordenite (Figures S6, S7 and Table S1).

**Figure 7:**
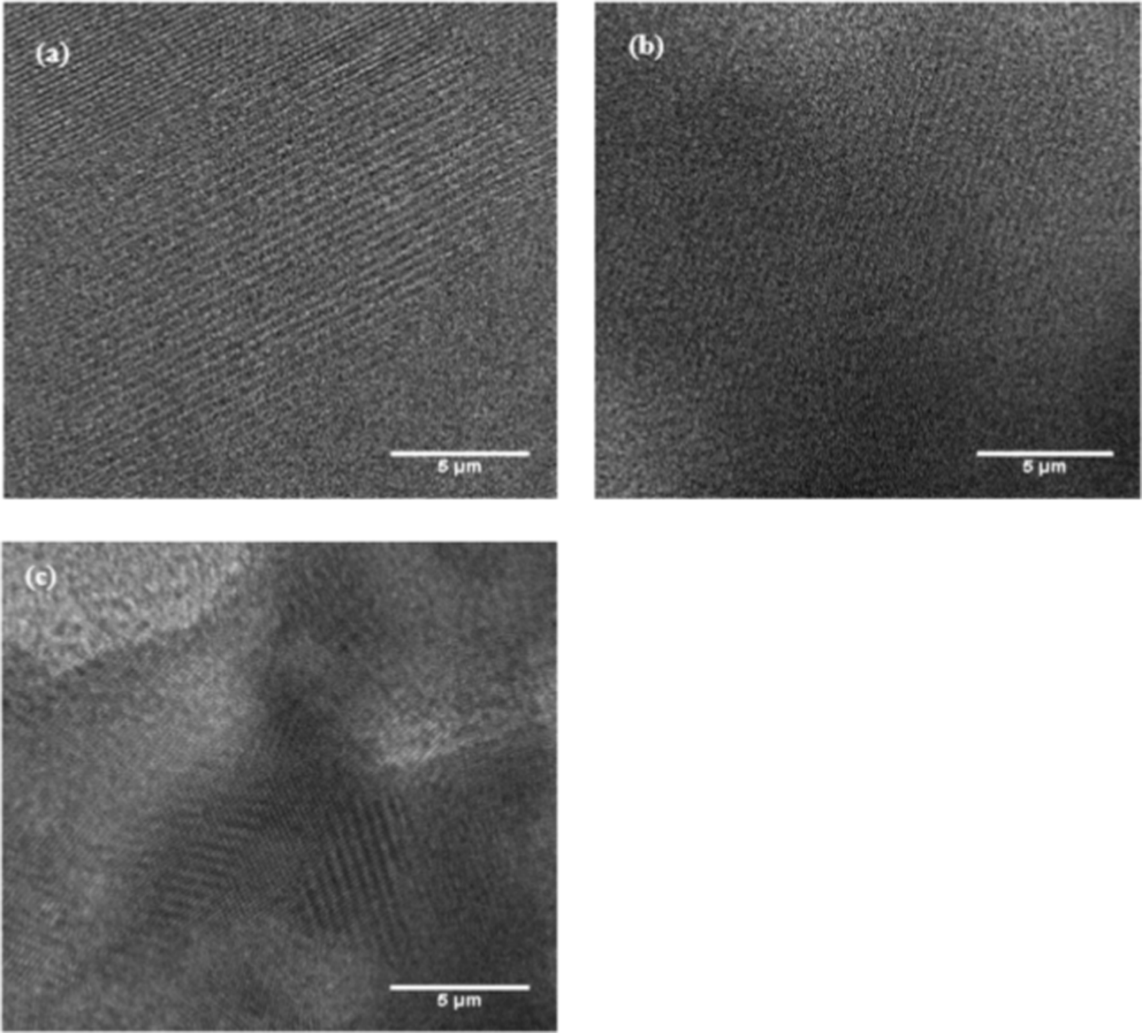
TEM images of: (a) HM; (b) Fe-HM; (c) Ru-HM Catalysts.

**Figure 8:**
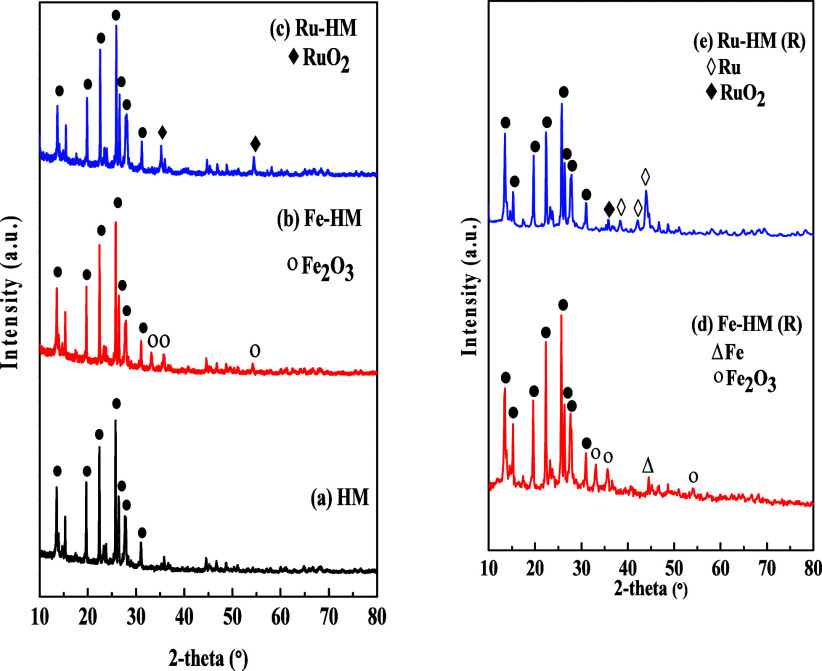
XRD analyses of Fresh (a) HM, (b) Fe-HM, and (c) Ru-HM and reduced (d) Fe-HM (R) and (e) Ru-HM (R) catalysts.

#### 3.1.6 XRD Analysis

XRD analysis was performed to determine the crystal size and crystallinity and to identify the structures of the synthesized catalysts, as shown in Figure 8. All catalysts exhibited almost the same XRD patterns with (111), (310), (330), (150), (202), (350), (132), and (402) planes at 2θ = 13.42°, 15.32°, 19.75°, 22.37°, 25.70°, 26.41°, 27.84°, and 30.93°, respectively (Figure 8a). The diffraction peaks correspond to the orthorhombic phase of the mordenite structure (JCPDS-43-0171).^17,36^ Small intense peaks of the Fe_2_O_3_ (hematite) phase of crystal are observed at 2θ = 33.33°, 35.71°, and 54.26° (Figure 8b). The corresponding diffraction planes are observed at (104), (110), and (116), respectively. The hematite crystal structure is Rhombohedral according to the JCPDS database (89-8103). In the case of the Ru-HM catalyst, two distinct peaks of tetragonal RuO_2_ are observed at 2θ = 35.27° and 54.54° that correspond to (101) and (211) crystal planes, respectively.^37,38^ The relative crystallinity of each sample was evaluated by analyzing all peak areas of crystals divided by the total peak area (crystal and amorphous). However, the crystallinity decreased slightly after incorporation of Fe_2_O_3_ and RuO_2_ in the HM support as shown in Table 3. Although the signal is relatively weak, Fe_2_O_3_ and RuO_2_ in HM zeolite exhibited this peak at higher angles compared to the parent HM. This shift is associated with the dealumination process, as the Si–O bond (1.60 Å) was shorter than the Al–O bond (1.74 Å) in mordenite. As the Al species are leached from the zeolite framework, the lattice contracts, suggesting successful removal of Al species.^39^ The crystal size was calculated using the modified Scherrer equation. The crystal size for the HM support is 44.46 nm. Similar crystal size was reported by Wen et al.^18^ They reported the HM crystal size varies between 40.3 and 45 nm. After Fe and Ru oxide incorporation, the intensity of the XRD signals decreased. These changes reflect the crystal sizes of HM for Fe-HM and Ru-HM catalysts. The crystal size of HM decreased a bit. The calculated crystal sizes of Fe-HM and Ru-HM are 43.33 and 42.64 nm, respectively. The Fe_2_O_3_ crystal size is 18.81 nm, whereas the RuO_2_ crystal size is 24.4 nm. As the RuO_2_ crystal size is bigger than that of Fe_2_O_3_, RuO_2_ may facilitate better heat transfer and gas–solid interactions, allowing reduction (RuO_2_ to Ru) to occur at lower temperatures. This is corroborated by H_2_-TPR studies (Section 3.1.7). The crystallite sizes estimated from XRD peak broadening reflect coherently diffracting domains of Fe_2_O_3_ and RuO_2_ (≈13−16 nm) and should not be interpreted as physical particle sizes. The SEM images show microscale secondary particles composed of aggregated nanoscale crystallites, a characteristic feature of metal-modified zeolite catalysts.

**Table 3:** Crystallinity and Crystal Sizes of All Catalysts

		crystal size (nm)^b^				
catalysts	crystallinity (%)^a^	Mordenite	Fe_2_O_3_	RuO_2_	Fe	Ru
HM	39.74	44.46				
Fe-HM	37.28	43.33	18.81		16.31	
Ru-HM	38.24	42.64		24.4		12.98

a $\%\;crystallinity=\frac{\text{all}\;crystals\;peak\;area}{total\;peak\;area\;\left(crystal+amorphous\right)}\times 100$ .

b Crystal sizes were calculated using the modified Scherrer equation.^42^

The XRD analysis of reduced catalysts (Fe-HM (R) and Ru-HM (R), where R represents reduced metals) was also performed to identify the reduced metals (Fe and Ru). The intense peaks of the Fe_2_O_3_ (hematite) phase of crystal are observed as in fresh catalyst (Figure 8d). The small peak at 2θ = 44.67° corresponds to the Fe phase (JCPDS-06-0696).^40^ In the Ru-HM (R) catalyst (Figure 8e), three distinct HCP (hexagonal closed packed) phases of Ru are observed at 2θ = 38.42°, 42.22°, and 44.13° that correspond to (100), (002), and (101) crystal planes, respectively.^41^ As discussed below in H_2_-TPR studies, the Ru-HM catalyst is easy to be reduced by H_2_ at lower temperature. Several Ru phase peaks are observed when they are compared to those of the Fe-modernite. The crystal sizes of Fe and Ru shrink after the reduction of the catalysts. The Fe crystal size is 16.31 nm, whereas the Ru crystal size is 12.98 nm.

#### 3.1.7 H_2_-TPR Analysis

The reduction of metal oxides and metal−support interaction was investigated by H_2_-TPR studies. The H_2_-TPR profiles of two Fe- and Ru-loaded catalysts are shown in Figure 9. In the case of the Fe-HM catalyst, three distinct peaks of Fe_2_O_3_ are observed at 367, 485, and 531 °C (Figure 9a). The formation of the hematite (Fe_2_O_3_) phase was also confirmed by XRD analysis. The reduction of Fe_2_O_3_ occurs in multiple steps that can be attributed to peaks in the TPR profile. The peaks correspond to the reduction of Fe_2_O_3_ phases with an increase in temperature in the following order: Fe_2_O_3_ (Hematite) → Fe_3_O_4_ (Magnetite) → FeO (wustite) → Fe.^43^ Reduction of Fe_2_O_3_ to Fe_3_O_4_ usually takes place at 320−380 °C (here, it is 367 °C). Reduction of Fe_3_O_4_ to FeO (wustite) is observed 485 °C. The formation of metallic Fe from the reduction of FeO generally occurs above 500 °C.^44,45^ From a thermodynamic point of view, iron oxide reduction can occur at a lower temperature. If the reduction temperature is lower than 570 °C, reduction occurs in two steps (Fe_2_O_3_ to Fe_3_O_4_ and Fe_3_O_4_ to α-Fe).^46,47^ In the case of the Ru-HM catalyst (Figure 9b), the reduction peak of RuO_2_ to metallic Ru is observed at 140 °C.^48^ The reduction peak of RuO_2_ at lower temperature reflects weaker metal−support interaction than that observed for the reduction of Fe_2_O_3_ in the presence of the same support (HM).

**Figure 9:**
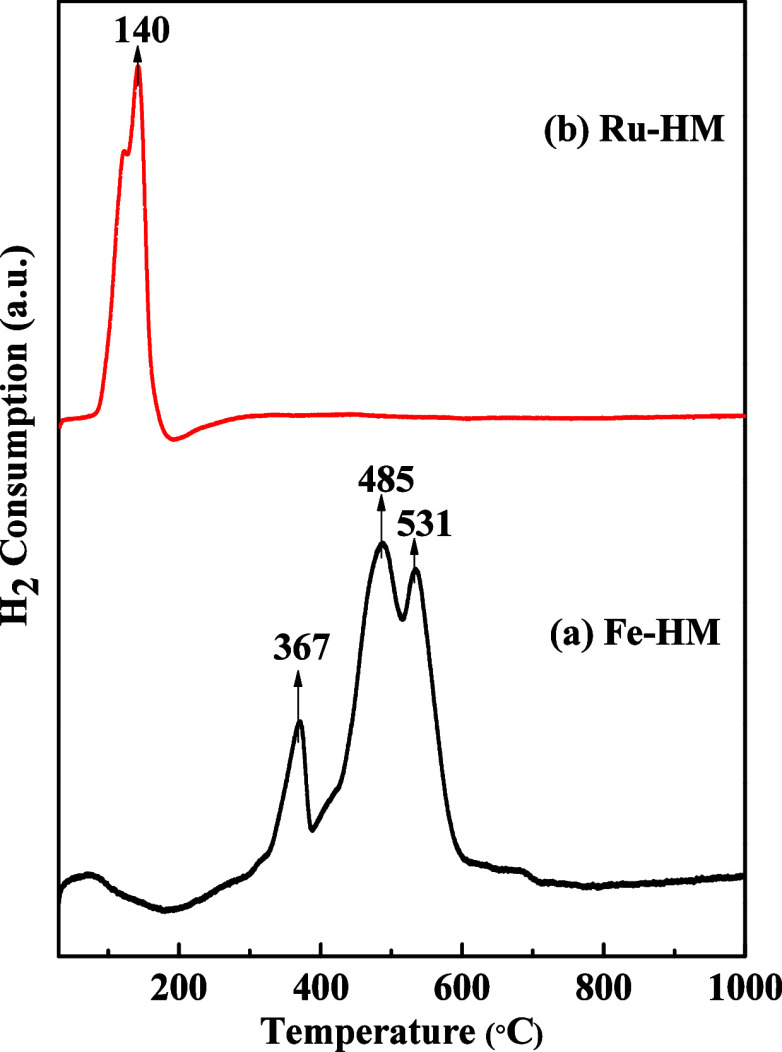
H_2_-TPR profiles of (a) Fe-HM; (b) Ru-HM catalysts.

H_2_ consumption by catalysts containing metal oxides yields reducible species that provide active sites for catalytic reactions. Table 4 shows the H_2_ consumption of both catalysts from H_2_-TPR studies. As higher H_2_ consumption is observed for Fe-HM catalysts, it shows the presence of a maximum number of reducible species. The H_2_ consumption of two catalysts is 931.9 and 460.6 μmol/g, respectively. Higher H_2_ consumption in Fe-HM suggests that Fe is present in a more oxidized form after the preparation of the catalyst. The H_2_ consumption data highlight the distinct redox behaviors of Fe-HM and Ru-HM, which directly influence their catalytic roles in PE pyrolysis. The higher H_2_ uptake of Fe-HM reflects greater redox cycling, enabling stronger hydrogen transfer that stabilizes radicals, enhances overall PE conversion, and suppresses coke formation. In contrast, the lower-temperature reducibility of Ru-HM facilitates faster hydrogen transfer, favoring selective production of light olefins, particularly propene (Table 4). Thus, Fe-HM primarily enhances stability and improves conversion, while Ru-HM enhances olefin selectivity.

**Table 4:** H_2_ Consumption by the Synthesized Catalysts

catalyst	H_2_ consumption (μmol/g)
Fe-HM	931.9
Ru-HM	460.6

#### 3.1.8 NH_3_-TPD Analysis of H-Modernite (HM), Fe-HM, and Ru-HM

Zeolites consist of structured SiO_4_ and AlO_4_ tetrahedral networks, where the isomorphous substitution of Al^3+^ for Si^4+^ creates negatively charged [AlO_4_]^−^ units, requiring extra-framework cations to maintain charge balance.^49^ The Brønsted acid sites (BAS) in zeolites originate from positively charged bridging hydroxyl groups (Si–OH–Al) when protons are present, whereas Lewis acid sites (LAS) arise from distorted extra-framework species, framework-associated aluminum, and tetra- or penta-coordinated aluminum within the structure.^49,50^

The acidity of the zeolite catalyst, including the strength of the acid sites, plays an important role in determining its efficacy for PE pyrolysis. The acid sites affect the catalytic pyrolysis process by controlling cracking and isomerization reactions.^51^ The Bronsted and Lewis acid sites of the H-mordenite (HM) zeolite and Fe-HM and Ru-HM were analyzed using NH_3_-TPD studies (Figure 10). NH_3_-TPD profiles of HM, Fe-HM, and Ru-HM (Figure 10) reveal that metal incorporation significantly modifies the total acidity and the distribution of weak and strong acid sites. It affects the breakdown of polymer chains and the selectivity of the pyrolysis products. The HM, Fe-HM, and Ru-HM samples show a nonsymmetrical NH_3_ desorption peak between 100 and 700 °C. The peak at lower temperature corresponds to desorption of physiosorbed NH_3_ by weak acid sites. The NH_3_ adsorption on the Bronsted acid sites and NH_3_ interaction with Si–OH groups should be attributed to the weak acid sites in the range of 100 to 290 °C.^17,52^ The high-temperature peak is attributed to NH_3_ absorbed by the acidic Al–OH at the interface. So, the intense peak between the temperature range of 300 to 700 °C is due to NH_3_ adsorption to the acidic sites of the Al framework.^53,54^ The negative charge of the zeolite framework is produced by the number of Al^3+^O_4_ units. When the negative charge is neutralized by a proton, it creates a particular area in the zeolite framework that works as a Bronsted acid site. This acid site acts as an active site in acid-catalyzed transformation to the products.^55,56^ The Bronsted acid sites in zeolites serve as the primary active centers for acid-catalyzed transformations, facilitating crucial steps, such as C–C bond cleavage, isomerization, and aromatization during the pyrolysis of polyolefins.

**Figure 10:**
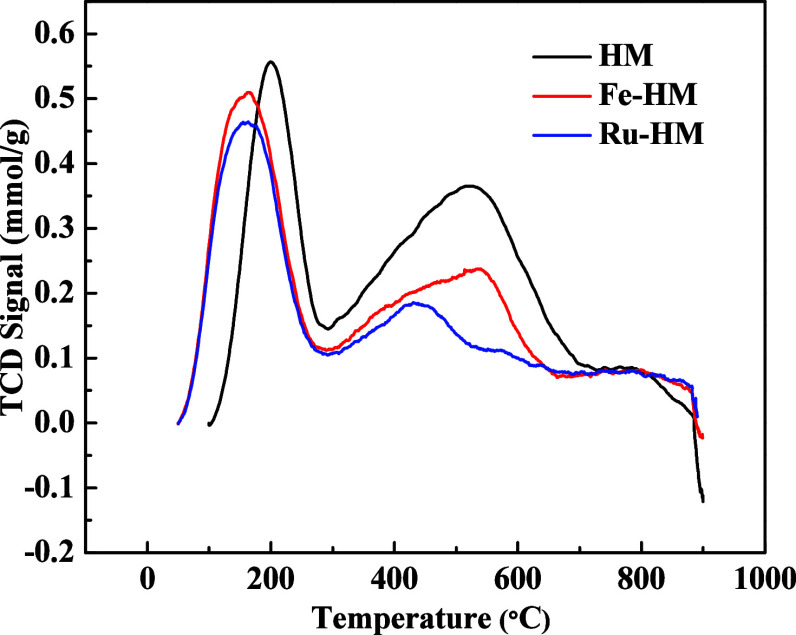
NH_3_-TPD analyses of H-Mordenite (HM) and metal-incorporated HM.

The concentration and strength of the acid sites in zeolites are largely governed by the silicon-to-aluminum (Si/Al) ratio of the framework. A lower Si/Al ratio corresponds to a higher aluminum content, which increases the density of Bronsted acid sites and thereby enhances catalytic cracking activity. Conversely, a higher Si/Al ratio reduces the number of acid sites but improves the hydrophobicity and thermal stability, which can favor selective production of hydrocarbon fractions. So, the Si/Al ratio plays a decisive role in dictating both the overall acidity and the catalytic performance of zeolites in plastic pyrolysis. Strong Bronsted acidic sites initiate the protonation of polymer chains, generating carbocation intermediates that undergo β-scission to yield smaller hydrocarbon fragments, thereby enhancing the cracking activity.

NH_3_-TPD measurements of HM, Fe-HM, and Ru-HM reveal systematic changes in the total acidity and acid strength distribution upon metal incorporation. While NH_3_-TPD does not differentiate between Bronsted and Lewis acid sites, the observed increase in medium-to-strong acidity for Fe-HM and Ru-HM is consistent with enhanced cracking and secondary reactions. Therefore, mechanistic interpretations are based on overall acidity modulation and metal−acid bifunctionality, with specific Bronsted and Lewis roles inferred from established literature^57,58^ rather than directly resolved experimentally.

Table 5 shows the low- and high-temperature peak acidity and total acidity of HM, Fe-HM, and Ru-HM. As shown in Table 5, the low-temperature peak acidity is higher. This indicates a greater abundance of weak to moderate acid sites. This result is consistent with more acidic sites belonging to the Si–OH group. The Si–OH groups participated in overall acidity and may influence catalytic activity by favoring milder cracking reactions.^59^ Incorporation of Fe and Ru into the HM framework increases the density of accessible acid sites, likely due to metal−framework interactions, partial dealumination, and the generation of additional Lewis acidic centers associated with metal oxide species. The higher HT acidity observed for Fe-HM reflects a greater population of strong acid sites, consistent with its higher aluminum-associated acidity and stronger metal−support interactions. In contrast, Ru-HM shows a slightly lower total acidity than Fe-HM but retains a balanced distribution of moderate and strong acid sites, which is favorable for selective cracking toward light olefins.

**Table 5:** Acidity Measurement of HM by NH_3_-TPD Analysis

catalyst	*LT peak (mmol/g)^a^	*HT peak (mmol/g)^a^	total acidity (mmol/g)^a^
HM	3.84	8.55	12.39
Fe-HM	8.41	7.16	15.57
Ru-HM	7.89	5.74	13.63

a Total acidity was determined by the standard temperature-programmed desorption of ammonia method. *LT = Low temperature; *HT = High temperature.

The Bronsted acid site (BAS) plays a crucial role in the pyrolysis of polyethylene, facilitating the formation of alkanes, alkenes, and aromatic hydrocarbons.^50,60,61^ Initially, plastic molecular chains adsorb onto the BAS, generating carbonium ion intermediates. Simultaneously, chain dehydrogenation produces unsaturated fragments, leading to the formation of carbenium ion intermediates, which then initiate the catalytic pyrolysis process^62^ to yield the observed products. The NH_3_-TPD analysis confirms that Fe-HM and Ru-HM possess significantly higher acidity than the parent HM, with distinct acid strength distributions that directly correlate with their observed catalytic behaviors during polyethylene pyrolysis. The enhanced strong acidity of Fe-HM favors higher PE conversion, while the moderated yet sufficient acidity of Ru-HM, combined with its superior hydrogen transfer capability, promotes selective formation of light olefins, particularly propene.

### 3.2 Thermo-Catalytic Deconstruction (Pyrolysis) of PE

Thermo-catalytic pyrolysis of polyethylene (PE) was carried out at three different reaction temperatures (400 °C, 450 °C, and 500 °C). The PE conversion and product distribution are listed in Figure 11. At 400 °C, approximately 73% of the PE conversion is observed. It increases to 82% and 81% at 450 and 500 °C, respectively. Although CO_2_ is the main product at 400 °C, its selectivity decreases with the increase of the pyrolysis temperature. The selectivity to paraffins and olefins also increases with the increase of temperature. The propane and propene selectivity mainly dominated at 450 °C. Ethylene selectivity considerably increased with that of propene at 500 °C. These results reflect that the olefins’ selectivity (ethylene and propene) increased with an increase in pyrolysis temperature.

**Figure 11:**
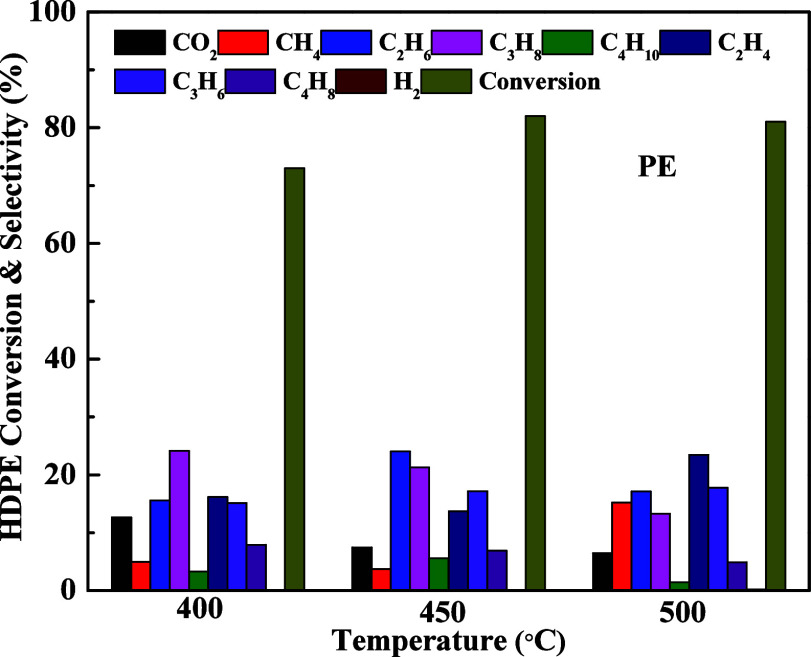
Pyrolysis of PE at different temperatures.

The effect of different catalysts on product selectivity and PE conversion was investigated by varying PE and catalyst weight ratios, as described in the next section.

Thermo-catalytic pyrolysis of PE in the presence of the H-mordenite (HM) catalyst with different PE to catalyst weight ratios at three different pyrolysis temperatures (400, 450, and 500 °C) is depicted in Figure 12a. A conversion of 76.2% of PE was obtained at 400 °C with a 1:1 weight ratio of PE and HM (Figure 12a). Similar PE conversions were observed at 450 and 500 °C. Almost the same conversion was recorded at 450 and 500 °C with a 1:2 weight ratio of PE to HM, where the conversion was 87.21% and 85.25%, respectively. A selectivity to propene ∼60% was observed at 400 °C (Figure 12a), which decreased at higher pyrolysis temperatures (450 and 500 °C). Paraffin (propane) selectivity mainly dominated at higher temperatures for a 1:1 PE to HM weight ratio. In the presence of a higher loading of the catalyst (HM), the propane selectivity is very high compared to those of other products (Figure 12b). Thus, the increased catalyst loading favors paraffin over olefin selectivity.

**Figure 12:**
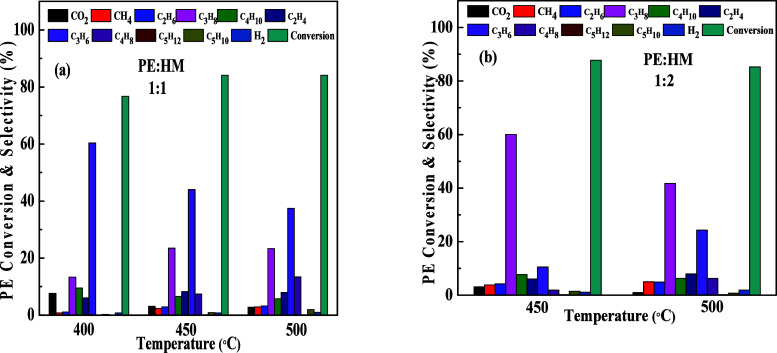
Thermo-catalytic pyrolysis of PE in the presence of H-M (HM) with different weight ratios, (a) 1:1; (b) 1:2 at different temperatures.

The effect of Fe-HM catalyst loading on the thermo-catalytic process (pyrolysis) was also investigated by varying temperatures (400 °C, 450 °C, and 500 °C) and weight ratios of PE and catalyst as shown in Figure 13a,b. With a 1:1 weight ratio of PE and catalyst, we observed a maximum PE conversion (95.86%) at 450 °C. The PE conversion was around 90.75% at 450 °C with a 1:2 weight ratio of PE to Fe-HM. In terms of product selectivity, a similar trend to HM was observed for Fe-HM catalysts. The selectivity to propane was initially high at 400 and 450 °C but decreased at 500 °C. In contrast, at 500 °C, propene selectivity (>40%) was higher compared to the paraffin selectivity. With a 1:2 weight ratio of PE and Fe-HM, the propane selectivity dominated at 450 and 500 °C. Propene selectivity increased slightly at 500 °C. Selectivity to paraffins increases with the increase in loading of the catalyst. As the selectivity of our product of interest (olefins) decreased with lower temperatures, we did not perform pyrolysis experiment at 400 °C for the 1:2 weight ratio of the PE to Fe-HM catalyst.

**Figure 13:**
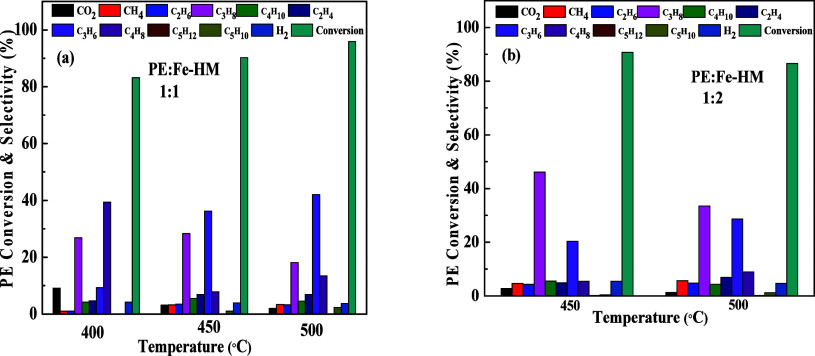
Thermo-catalytic pyrolysis of PE in the presence of Fe-HM with different weight ratios: (a) 1:1; (b) 1:2 at different temperatures.

Thermo-catalysis of PE in the presence of the Ru-loaded HM catalyst was also investigated with different PE to Ru-HM catalyst weight ratios, as shown in Figure 14. PE conversion increased with the increase of pyrolysis temperature for the 1:1 weight ratio. The conversion was 88.08, 90.27, and 96.81% for 400 °C, 450 °C, and 500 °C, respectively. Similar PE conversions were achieved at both temperatures (450 and 500 °C) with a 1:2 PE to Ru-HM weight ratio. The conversion was 89.84 and 90.19% at 450 and 500 °C, respectively. Propene (olefin) selectivity increased with the increase in temperature (Figure 14a). More than 40% selectivity of propene was achieved at 500 °C. However, when the weight ratio of the PE to Ru-HM catalyst (Figure 14b) was increased to 1:2, the propane selectivity dominated. A selectivity of 60% propane (for both cases 1:1 and 1:2 ratios) was obtained at 400 and 450 °C, respectively. As the desired product (olefins) selectivity decreased with the decrease in temperature, we did not perform the pyrolysis experiment at 400 °C for the1:2 weight ratio of the PE to Ru-HM catalyst. The reaction rate of pyrolysis is calculated based on the 1:1 weight ratio of the PE to Ru-HM catalyst. The rate values are 2.17 × 10^−10^, 2.22 × 10^−10^, and 2.38 × 10^−10^ mol·g^−1^·s^−1^ at 400, 450, and 500 °C, respectively. The complete mass balance of thermo-catalytic pyrolysis results is shown in Tables S2−S8 in the Supporting Information.

**Figure 14:**
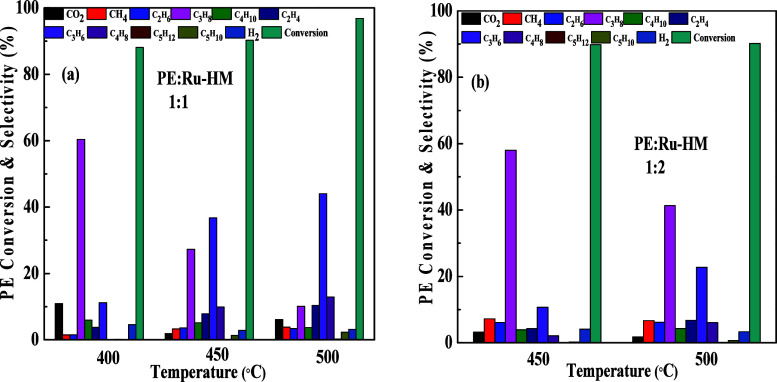
Thermo-catalysis of PE in the presence of Ru-HM with different weight ratios (a) 1:1; (b) 1:2 at different temperatures.

### 3.3 Mechanistic Aspects of Thermo-Catalytic Pyrolysis of PE

The FTIR spectra confirmed that the zeolite framework remained intact after metal impregnation, with decreased peak intensities suggesting surface deposition of Fe and Ru without significant structural collapse. The XRD analysis further supports this observation, showing only a slight decrease in crystallinity after metal incorporation. Fe_2_O_3_ crystallites (∼18.8 nm) and RuO_2_ crystallites (∼24.4 nm) are well dispersed, with the larger RuO_2_ particles potentially facilitating faster reduction and hydrogen transfer. Our XPS studies confirm the coexistence of Fe^2+^/Fe^3+^ species in Fe-HM and Ru^4+^ species in Ru-HM before reduction. In postreduction, metallic Fe° and Ru° phases appear, which are active for hydrogenation–dehydrogenation reactions during pyrolysis. SEM-EDS demonstrated uniform metal dispersion, while TEM images confirmed preservation of crystalline ordering in HM after metal impregnation.

BET analyses reveal that incorporation of Fe and Ru into HM slightly reduced the surface area due to partial pore blockage; however, mesoporosity is retained, ensuring effective diffusion of PE vapors to active sites. The presence of both micro- and mesopores (H4-type hysteresis) likely enhances secondary cracking of heavier fragments into light olefins as discussed below. The strong acidic sites of zeolite-based catalysts (HM) play a crucial role in facilitating the dehydrogenation of short-chain alkanes, leading to increased olefin production.^63^

Mechanistically, thermal pyrolysis of polyethylene (PE) occurs through a free radical mechanism that involves random and chain-end scission, followed by chain recombination.^64^ In contrast, catalytic pyrolysis follows a carbocation chain mechanism, facilitated by solid acid catalysts like zeolites, and includes secondary reactions such as isomerization, cyclization, and dehydrogenation.^49^

The analysis of gaseous products, both with and without catalysts, provides key insights into the thermo-catalytic pyrolysis mechanism of polyethylene (PE). The gaseous fraction consists primarily of light olefins (C_2_H_4_, C_3_H_6_), followed by short-chain alkanes (CH_4_, C_2_H_6_, C_3_H_8_, and C_4_H_10_), CO_2_, and H_2_. This distribution is observed using HM, Fe-HM, and Ru-HM catalysts at a consistent PE-to-catalyst weight ratio of 1:1 across all reaction temperatures.

Incorporation of Fe and Ru into the zeolite framework further enhanced the catalytic activity, promoting the formation of olefins and H_2_. The hydrogenation capability of reduced metals aids in stabilizing free radicals formed during pyrolysis, thereby promoting the generation of aliphatic hydrocarbons. Their efficient hydrogen transfer facilitates the breakdown of macromolecules into aliphatic hydrocarbons.^65^ CO_2_ production primarily results from oxygen removal via decarbonylation and decarboxylation reactions,^66^ while H_2_ generation was associated with the dehydrogenation of short-chain olefins.^67^ As the catalytic pyrolysis experiments were conducted using prereduced catalysts and polyethylene is oxygen-free, CO_2_ formation cannot be attributed to oxygen removal from the polymer backbone or to bulk lattice oxygen of metal oxides. Instead, CO_2_ likely originates from reactions involving residual surface oxygen species, zeolite framework hydroxyl groups, trace oxygen, or moisture impurities, and secondary oxidation of CO intermediates, with their contributions becoming more pronounced at elevated temperatures.

H_2_-TPR data clearly showed higher H_2_ consumption for Fe-HM (931.9 μmol/g) compared to Ru-HM (460.6 μmol/g), indicating a greater abundance of reducible species for Fe. Fe_2_O_3_ underwent stepwise reduction (Fe_2_O_3_ → Fe_3_O_4_ → FeO → Fe°), whereas RuO_2_ is reduced at a much lower temperature (∼140 °C), reflecting weaker metal−support interactions. The easier reducibility of RuO_2_ likely enhances hydrogen transfer reactions, which probably contributes to the higher propene selectivity observed at 500 °C.

Building on these redox characteristics, the structural integrity of the zeolite framework becomes equally critical in dictating the catalytic performance. From a mechanistic standpoint, the strong Brønsted acid sites in HM catalyze the adsorption of PE chains and their cleavage into carbocation intermediates, initiating β-scission to produce light olefins. Weak and moderate acid sites promote isomerization and selective cracking, whereas metal sites from Fe_2_O_3_ and RuO_2_ (and their reduced forms) enhance hydrogen transfer, stabilize radicals, and suppress excessive coke formation. CO_2_ generation occurred mainly via decarbonylation/decarboxylation, while H_2_ evolution was linked to the dehydrogenation of olefins.

Reaction temperature strongly influences product distribution, with higher temperatures favoring increased olefin and H_2_ yields due to enhanced cracking and dehydrogenation rates. A higher catalyst loading provided more available acid sites for PE vapor, which intensified secondary cracking and increased coke deposition.^68^ Thermogravimetric analysis (TGA) confirmed higher coke amounts at a PE-to-catalyst weight ratio of 1:2 (Figures S3−S5), likely due to prolonged residence time of PE vapor caused by mass transfer limitations.^69^ Despite this overall, PE conversion remained relatively stable with increased catalyst loading, suggesting minimal impact on total conversion.^62^

In order to have a better understanding of catalytic pyrolysis of PE, a DFT study was performed for PE in the presence of a Ru-HM catalyst. This provides mechanistic insights into the elementary steps of chain scission and hydrogen transfer. The theoretical results support the experimental findings, confirming that Ru sites lower the activation barriers for hydrogen transfer and radical stabilization, thereby rationalizing the enhanced olefin selectivity. A proposed mechanism on thermo-catalytic pyrolysis of polyethylene (PE) is shown in Figure 15. As illustrated in Figure 15, polyethylene is first thermally decomposed in a noncatalytic pyrolysis zone, producing PE radicals that are subsequently upgraded over an ex situ metal-modified H-mordenite catalyst bed. Thus, all catalytic transformations discussed here occur in the vapor phase and not via direct contact between molten PE and the catalyst.

**Figure 15:**
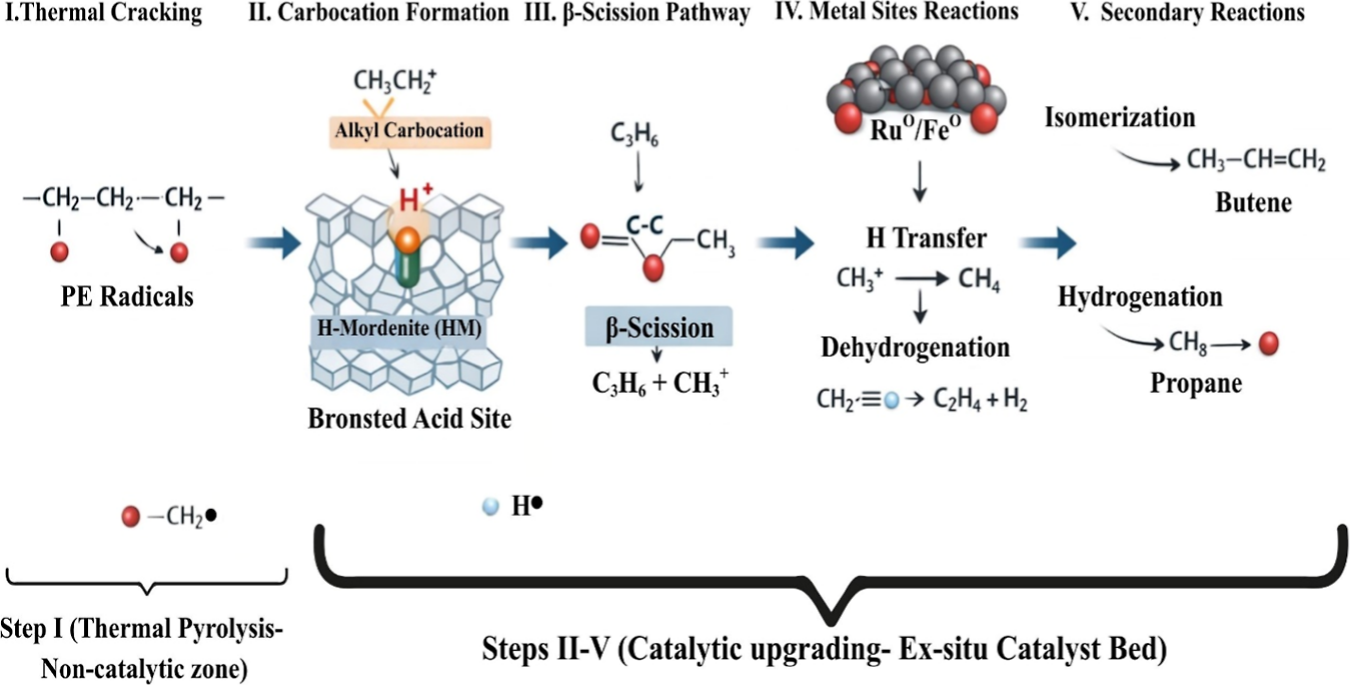
Proposed mechanism on thermo-catalytic pyrolysis of polyethylene (PE).

### 3.4 Mechanistic Aspects of Thermo-Catalytic Pyrolysis of PE via DFT Studies

Using the DFT-derived framework, machine-learning-enabled thermochemistry estimator (METE),^70^ we demonstrate the mechanistic pathways of pyrolysis of C_4_ on Ru active centers. We surveyed the C_4_ molecule as a surrogate of polyethylene, ensuring a balance between physicality and computational tractability. We considered C4 as our surrogate molecule, as it features the minimal carbon backbone to allow cracking at the terminal and middle positions. It is noteworthy that the current model empowers us to study the aspects of cracking locations and their implications, while it does not provide complicated aspects, including long-chain β-scission, radical propagation, or entropic effects, requiring more advanced models and dedicated computational work. Figure 16 demonstrates the most competitive reaction routes leading to terminal cracking and their corresponding free energies of stable and transition (BEP relation)^71^ species, all referencing to their standard heat of formation to NIST standards. From the figure, we observe that intrinsically, early-stage cracking is prohibitive, as shown in the fourth red tile in the graph, whereas late-stage cracking is preferred over dehydrogenation, which is in line with previous work.^72,73^ The energy span (the energy gap between the highest and lowest levels) can be used in an energy span model to estimate the turnover frequency (TOF) without needing a full microkinetic model,^74^ with applied thermal correction such as entropic contributions.^75^ It is calculated that at 500 °C, energy span shrinks significantly to 29.22 kcal/mol, giving a TOF of 5.55 s^−1^ (*k*_B_ *T*/*h* = 1 × 10^9^ s^−1^) and a corresponding reaction rate of 7.309 × 10^−9^ mol·g^−1^ per 5 wt % Ru catalyst when converted to a PE basis (MW = 376,270 g mol^−1^). Details of the calculation are available in the Supporting Information.

**Figure 16:**
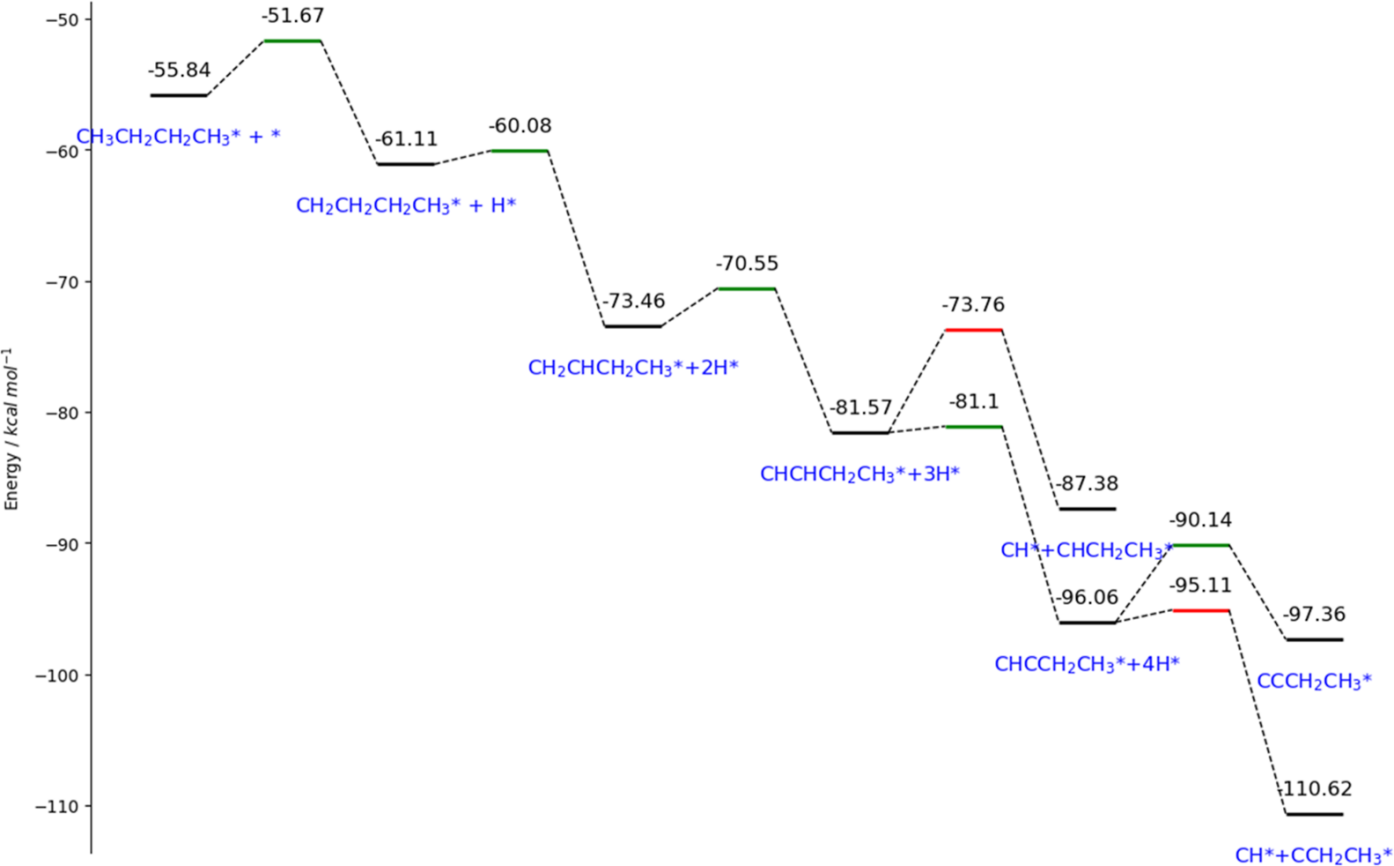
Energy diagram showing the dominant mechanism of C4 (surrogate molecule) cracking at the terminal position on the Ru (0001) surface. Color codes: Black, stable species; Green, transition states of dehydrogenation; Red, transition states of cracking.

We also explored the mechanism in relation to middle chain cracking. Similar to terminal cracking, early-stage cracking is not favored, as demonstrated by the prohibitively high barrier on the fourth TS tile marked in red in Figure 17. Once the surface species reaches deep dehydrogenation, namely, after 4 steps, cracking becomes possible. In fact, it is more favored to proceed than extremely deep dehydrogenation (which may lead to coking). This pathway yields ethane precursors, which are more valuable and therefore more desired over methane, the main product from the previous route in Figure 16. At 500 °C, the energy span shrinks only moderately to 38.13 kcal/mol, leading to a sluggish TOF of 0.016 s^−1^ (*k*_B_ *T*/*h* = 1 × 10^9^ s^−1^) and a corresponding reaction rate of 2.103 × 10^−11^ mol·g^−1^ per 5 wt % Ru catalyst when converted to a PE basis. Details of the calculation are available in the Supporting Information.

**Figure 17:**
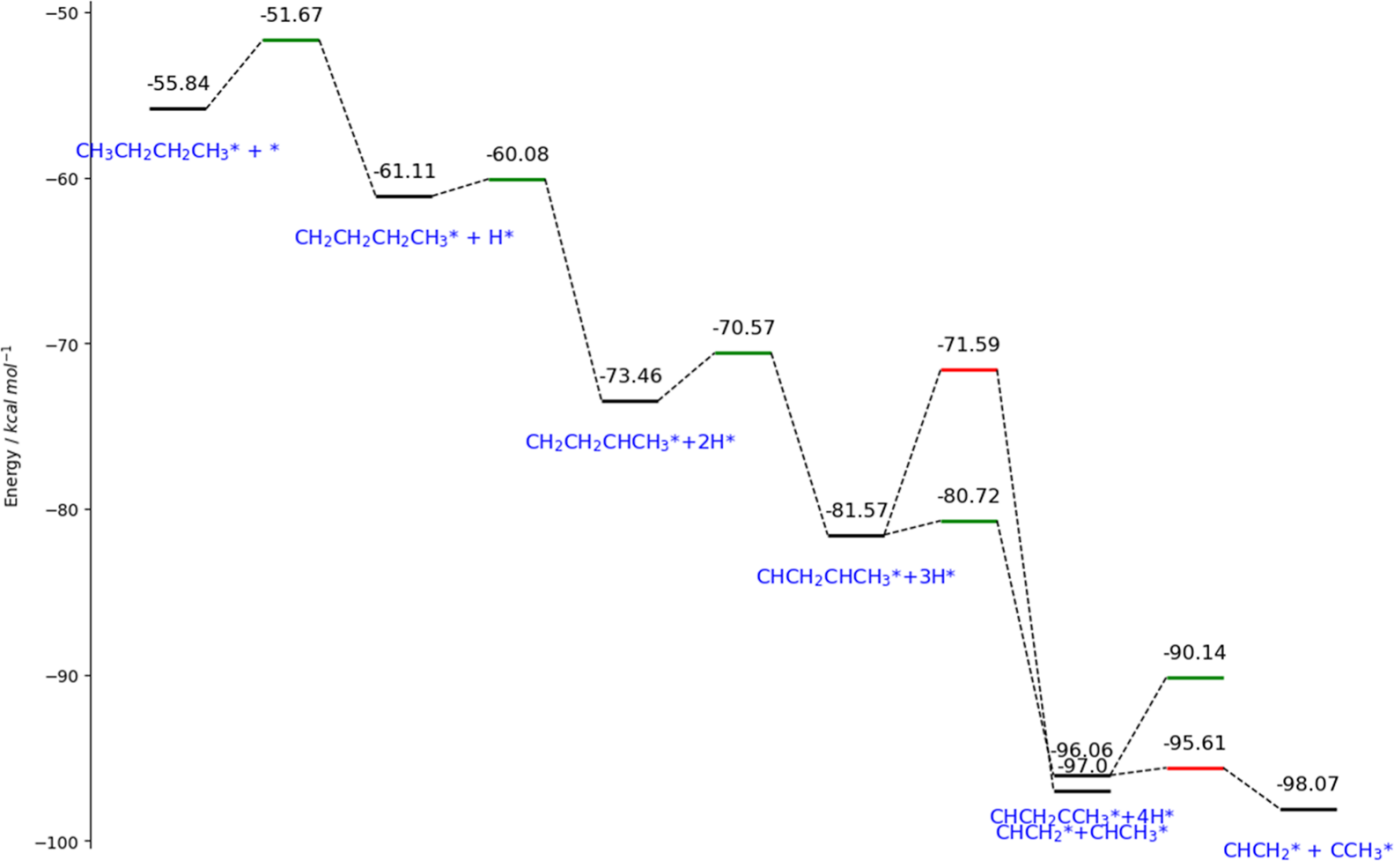
Energy diagram showing the dominant mechanism of C4 (surrogate molecule) cracking at the middle position on the Ru (0001) surface. Color codes: Black, stable species; Green, transition states of dehydrogenation; Red, transition states of cracking.

## 4 Conclusions

This study demonstrates the use of Fe- and Ru-based H-mordenite (HM) zeolite catalysts in the thermo-catalytic pyrolysis of polyethylene (PE) to produce light olefins and alkanes. The catalysts were systematically characterized to evaluate their textural, acidic, and structural properties. Results from BET, NH_3_-TPD, H_2_-TPR, and FTIR analyses highlight the influence of metal impregnation on the HM support’s catalytic activity. The pyrolysis experiments reveal that the temperature and polymer-to-catalyst weight ratios significantly influence the distribution of gaseous products. While propene selectivity exceeds 40% with Ru-HM, Fe-HM yielded a slightly lower propene selectivity under similar conditions. Increasing the pyrolysis temperature, in general, enhances olefin production with notable selectivity for ethylene and propene at 500 °C. Moreover, higher catalyst loading favors paraffin formation, indicating a trade-off between olefin and paraffin selectivity depending on the reaction conditions. Based on our DFT calculations, we conclude that cracking in the middle chain intrinsically has a lower TOF than cracking at the terminal on Ru(0001) at 500 °C. It is interesting that the experimental rate on Ru at 500 °C, 2.38 × 10^−10^ mol·g^−1^·s^−1^ falls under the computational predicted extreme bounds, 2.103 × 10^−11^ mol·g^−1^·s^−1^ to 7.309 × 10^−9^ mol·g^−1^·s^−1^. A naïve distribution would imply 97% carbon following cleavage at the middle chain position. We propose that this altered catalyst function (likely from support) essentially preserves the integrity of the median carbon backbone, thus yielding more desirable products. The outcome is distinctive than the ones from conventional Ru catalysts, which are known for facilitating cascade reactions via terminal cracking, result in a noticeable amount of undesired C_1_ species at elevated temperatures.^76,77^ Our study provides a vital roadmap for sustainable PE waste management by demonstrating that rational catalyst design and process optimization are the keys to effective, efficient, and high-value plastic-to-fuel conversion.

## Supplementary Material



## Notes

SEM-EDS images of the Fe-HM catalyst (Figure S1); SEM-EDS images of the Fe-HM catalyst (Figure S2); TGA analysis of spent catalysts with varying PE to HM weight ratios (Figure S3); TGA analysis of spent catalysts with varying PE to Fe-HM weight ratios (Figure S4); TGA analysis of spent catalysts with varying PE to Ru-HM weight ratios (Figure S5); TEM-EDS images of the Fe-HM catalyst (Figure S6); TEM-EDS images of the Ru-HM catalyst (Figure S7); TEM-EDS results of Fe-HM and Ru-HM catalysts (Table S1) and their discussion; apparent reaction rate calculation; mass balance for thermo-catalytic pyrolysis of PE in the presence of HM, Fe-HM, and Ru-HM catalysts at different temperatures (Tables S2–S8); DFT studies; entries for reaction mechanism; probed reactions with calculated formation enthalpies (Table S9); and calculations using the energy span model (PDF)
