# Supplementary material for: Fe- and Ru-H-Mordenites for Polyethylene Upcycling: Insights from Thermo-Catalytic Pyrolysis and DFT Studies
Source: ACS Sustain Chem Eng. 2026 Mar 30;14(16):7612–26. doi: 10.1021/acssuschemeng.5c11349 (PMC13126688; doi:10.1021/acssuschemeng.5c11349)
Supplement: Supplementary file 1 [file sc5c11349_si_001.pdf]

**Supporting Information**

# Fe- and Ru-H-Mordenites for Polyethylene Upcycling: Insights from Thermo-Catalytic Pyrolysis and DFT Studies

Gita Pandey [a]#, Sujoy Bepari [a]#, Sumit Gupta[a], Tianjun Xie[c], Debasish Kuila [a, b]*

[a] Department of Chemistry, North Carolina Agricultural and Technical State University, Greensboro, North Carolina, 27411, United States

[b] Joint School of Nanoscience and Nanoengineering, North Carolina Agricultural and Technical State University, Greensboro, NC 27411, USA

[c] Department of Chemical, Biological and Bioengineering, North Carolina Agricultural and Technical State University, Greensboro, North Carolina, 27411, United States

*Corresponding author: dkuila@ncat.edu

#equal contributions

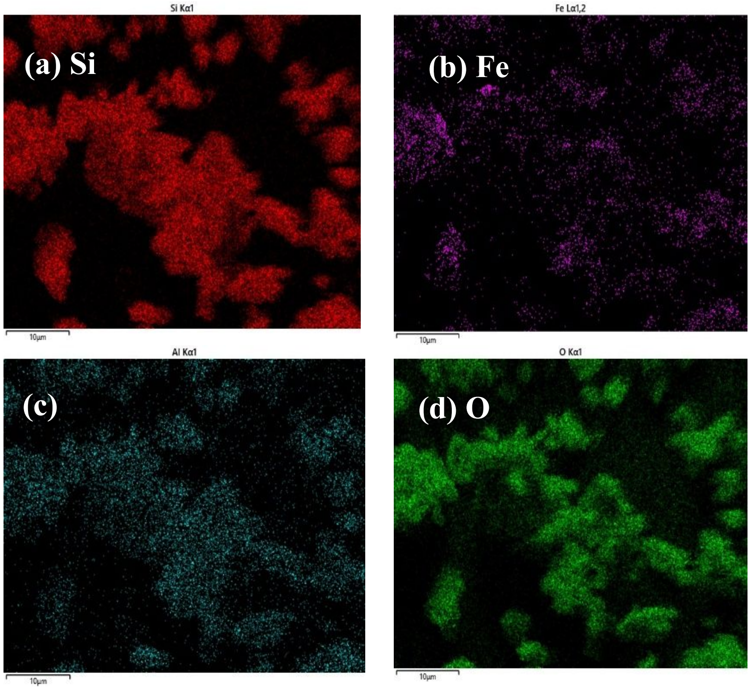


**Figure S1. SEM-EDS images of Fe-HM catalyst: (a) Si; (b) Fe; (c) Al; (d) O**

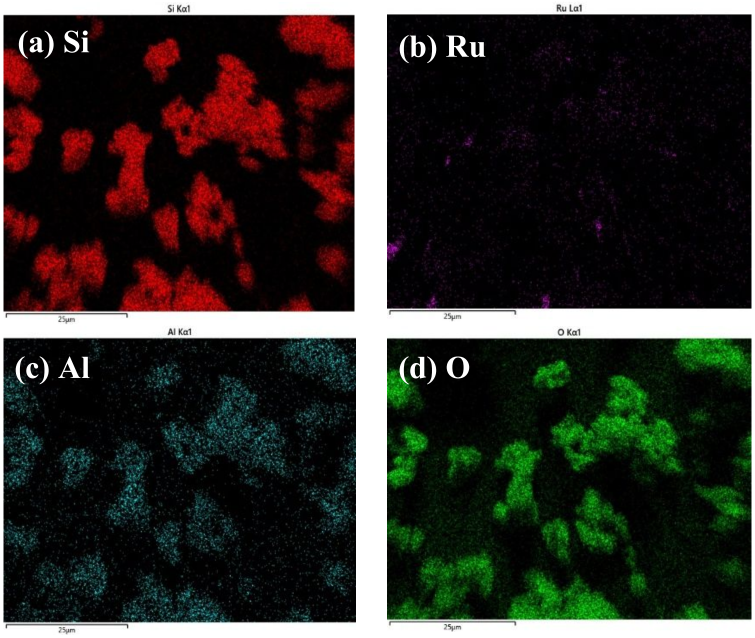


**Figure S2. SEM-EDS images of Ru-HM catalyst: (a) Si; (b) Ru; (c) Al; (d) O**

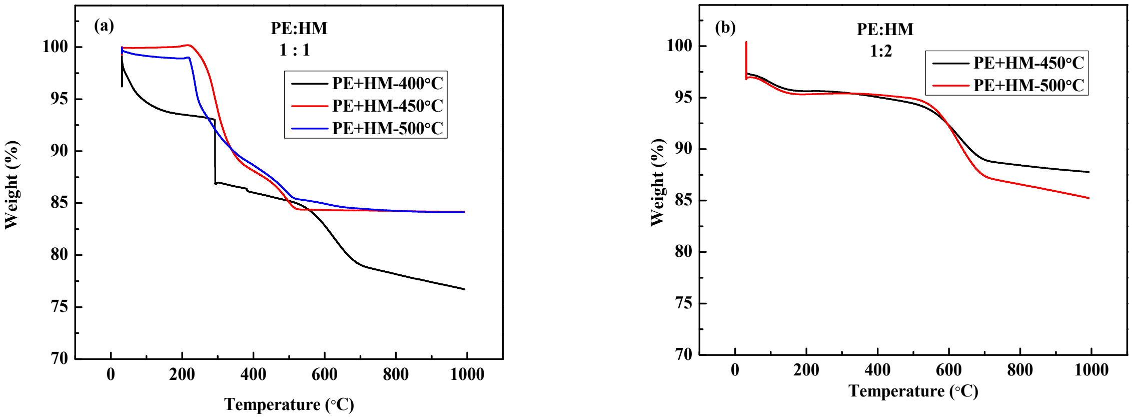


**Figure S3. TGA analyses of spent catalysts with varying PE to HM weight ratios: (a) 1:1 (b) 1:2**

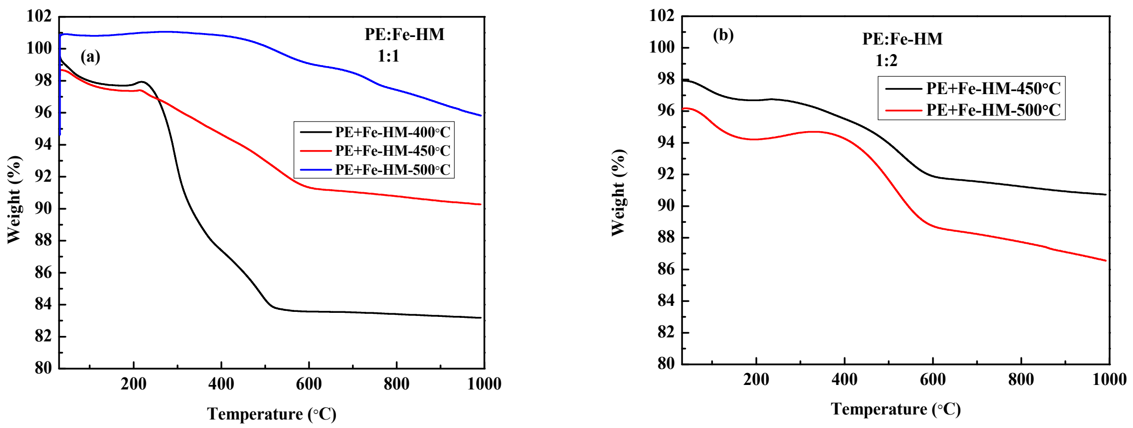


**Figure S4. TGA analyses of spent catalysts with varying PE to Fe-HM weight ratios: (a) 1:1 (b) 1:2**

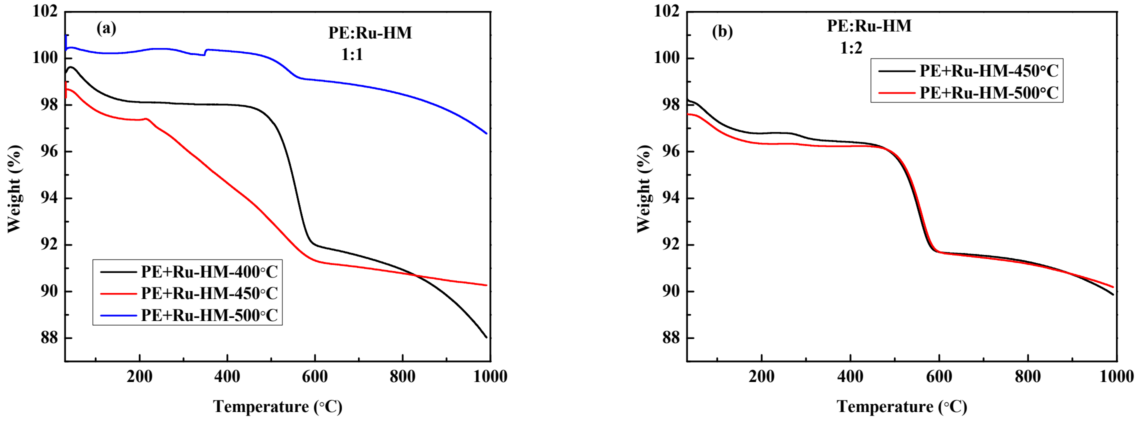


**Figure S5. TGA analyses of spent catalysts with varying PE to Ru-HM weight ratios: (a) 1:1 (b) 1:2**

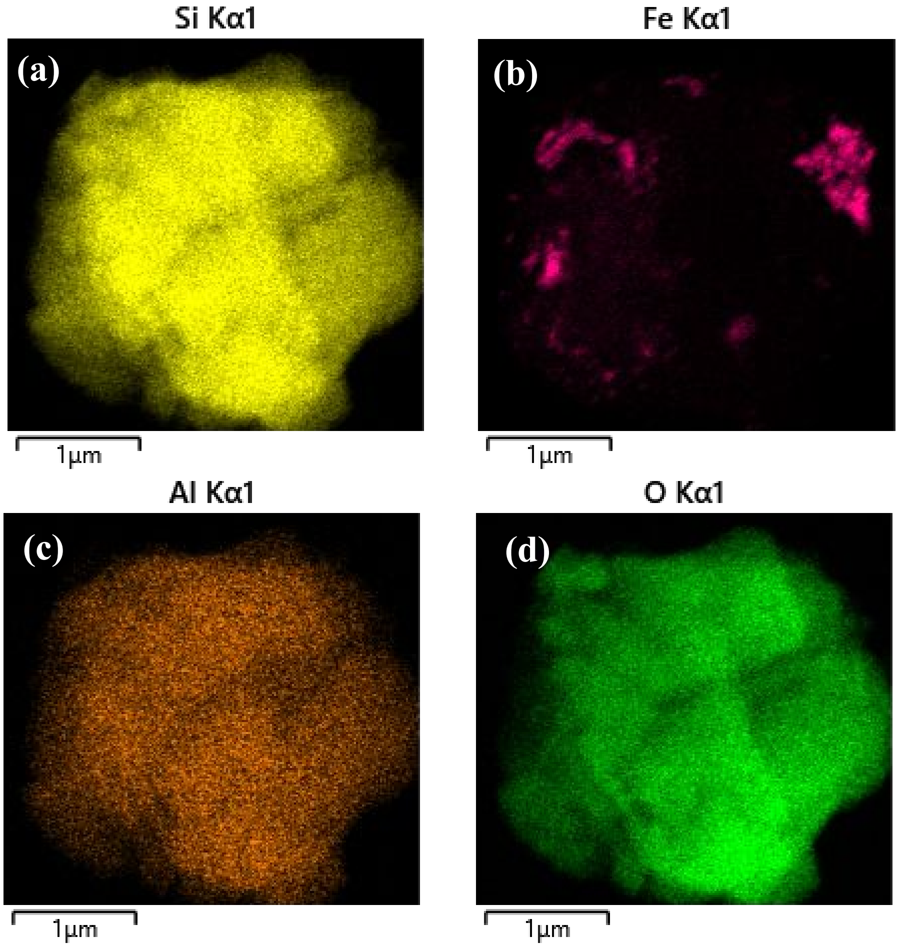


**Figure S6. TEM-EDS images of Fe-HM catalyst: (a) Si; (b) Fe; (c) Al; (d) O**

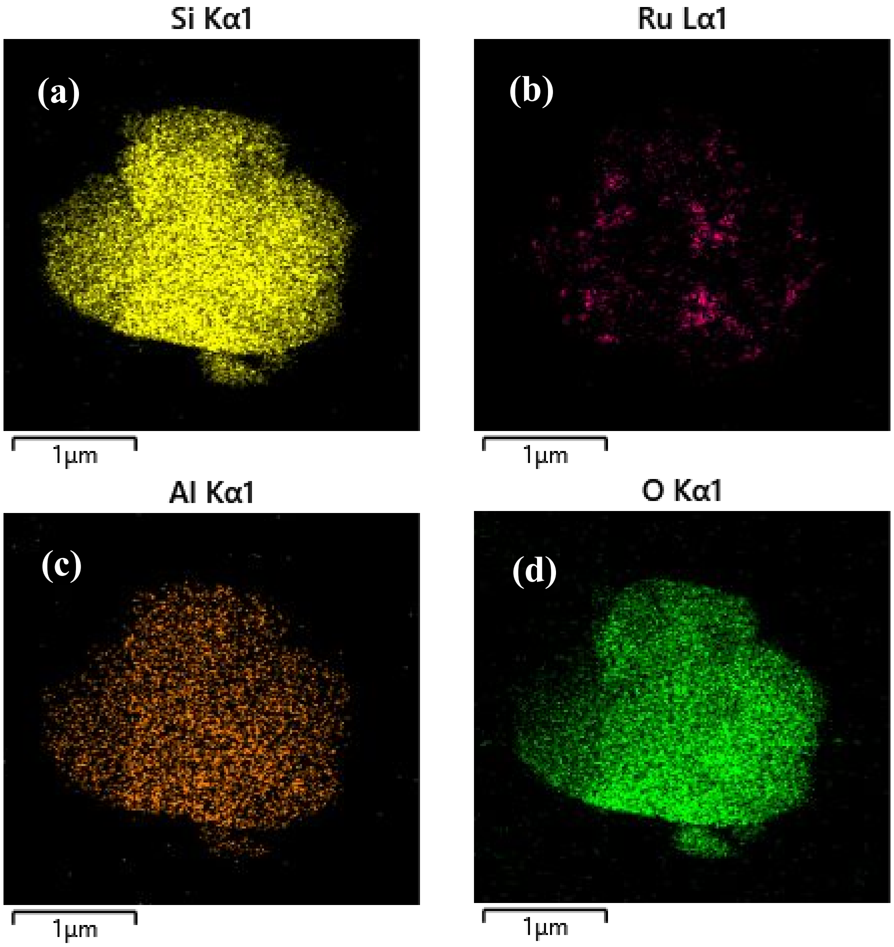


**Figure S7. TEM-EDS images of Ru-HM catalyst: (a) Si; (b) Ru; (c) Al; (d) O**

**Table S1. TEM-EDS results of Fe-HM and Ru-HM catalysts**

| Catalyst | Loading (wt.%) | | | | |
|---|---|---|---|---|---|
| | Si | Al | Fe | Ru | O |
| Fe-HM | 53.6 | 4.6 | 3.7 | - | 38 |
| Ru-HM | 46.9 | 5.8 | - | 4.7 | 42.6 |

The Si/Al ratios reported in Table S1 (11.65 for Fe-HM and 8.086 for Ru-HM) were obtained from local TEM–EDS measurements, which probe a very small, near-surface region of individual zeolite crystallites rather than the bulk composition. These values should be interpreted as local Si/Al ratios, not absolute bulk framework ratios[1]. The observed difference primarily originates from the different metal incorporation routes and metal–support interactions during catalyst preparation.

More significantly, this variation in local Si/Al ratio is not the dominant factor governing the distinct catalytic performances of Fe-HM and Ru-HM as bulk acidity trends (from $NH_3$-TPD/acid site analysis), discussed in the manuscript, remain comparable, and no systematic correlation between local Si/Al ratio and activity/selectivity is observed[2]. Instead, the performance differences are primarily attributed to:
(i) the intrinsic catalytic roles of Fe versus Ru,
(ii) differences in metal distribution and oxidation state, and
(iii) metal–acid site synergy, which controls reactant activation and product selectivity.

Thus, while the TEM–EDS-derived Si/Al ratios reflect local compositional variations induced by metal loading, they do not imply a fundamental change in the zeolite framework or acidity sufficient to explain the observed catalytic trends.

References:


1. W. Wan, J. Su, X.D. Zou, T. Willhammer. Transmission electrom microscopy as an important tool for characterization of zeolite structures. Inorg. Chem. Front. 2018, 5, 2836-2855. DOI: 10.1039/C8QI00806J.

2. E.G. Derouane, J.C. Vedrine, R. Ramos Pinto, P.M. Borges, L. Costa, M.A.N.D.A. Lemos, F. Ramoa Ribeiro. The Acidity of Zeolites: Concepts, Measurements and Relation to Catalysis: A Review on Experimental and Theoretical Methods for the Study of Zeolite Acidity. Catalysis Review, 2013, 454-515. DOI: 10.1080/01614940.2013.822266


**Apparent reaction rate calculation**

The apparent reaction rate for polyethylene (PE) pyrolysis was estimated based on the overall rate of PE conversion to gaseous products under steady operating conditions. For consistency and to minimize mass-transfer effects, the rate analysis was performed using a fixed PE-to-catalyst weight ratio of 1:1 over the Ru-HM catalyst.

The apparent reaction rate ($r_{app}$) was calculated using the following expression:

$$r_{app} = \frac{n_{PE,conv}}{m_{cat}.t}$$

where

- $n_{PE,conv}$ is the number of moles of polyethylene converted during the reaction (mol),
- $m_{cat}$ is the mass of catalyst used (g), and

- $t$ is the effective reaction time (s).

The moles of converted polyethylene were determined from gravimetric measurements:

$$n_{PE,conv} = \frac{p_i - p_o}{M_{PE}}$$

where

- $p_i$ and $p_o$ are the initial and final weights of PE (g), respectively, and
- $M_{PE}$ is the average molecular weight of the PE feed (376,270 g $mol^{-1}$, based on supplier (Dow chemicals) specifications.

The effective reaction time corresponds to the isothermal hold period at the target pyrolysis temperature, during which gas evolution reached a steady state as monitored by online GC-MS.

The calculated apparent reaction rates for Ru-HM were:

- $2.17 \times 10^{-10}$ mol $g^{-1}$ $s^{-1}$ at 400 °C,
- $2.22 \times 10^{-10}$ mol $g^{-1}$ $s^{-1}$ at 450 °C, and
- $2.38 \times 10^{-10}$ mol $g^{-1}$ $s^{-1}$ at 500 °C.

**Mass balance for Thermo-catalytic pyrolysis of PE in presence of HM, Fe-HM and Ru-HM catalysts at different temperatures**

**Table S2**. Pyrolysis of PE at different temperatures

| Temperature ($^0$C) | 400 | | 450 | | 500 | |
|---|---|---|---|---|---|---|
| | Gas (%) | Solid (%) | Gas (%) | Solid (%) | Gas (%) | Solid (%) |
| Product gases | 74.12 | 25.88 | 82.22 | 17.78 | 80.95 | 19.05 |
| $CH_4$ | 3.69 | | 3.06 | | 2.72 | |
| $CO_2$ | 9.37 | | 6.15 | | 4.41 | |
| $C_2H_4$ | 11.99 | | 11.29 | | 19.46 | |
| $C_2H_6$ | 11.58 | | 19.81 | | 19.16 | |
| $C_3H_6$ | 11.21 | | 14.14 | | 11.82 | |
| $C_3H_8$ | 17.93 | | 17.5 | | 12.25 | |
| $C_4H_{10}$ | 2.46 | | 4.62 | | 10.63 | |
| $C_4H_8$ | 5.85 | | 5.64 | | 0.49 | |
| $H_2$ | 0.04 | | 0.01 | | 0.01 | |

**Table S3.** Thermo-catalytic pyrolysis of PE in presence of HM with weight ratio (1:1) at different temperatures

| Temperature ($^0C$) | 400 | | 450 | | 500 | |
|---|---|---|---|---|---|---|
| | Gas (%) | Solid (%) | Gas (%) | Solid (%) | Gas (%) | Solid (%) |
| Product gases | 76.71 | 23.29 | 84.11 | 15.89 | 84.11 | 15.89 |
| $CH_4$ | 0.14 | | 0.43 | | 0.52 | |
| $CO_2$ | 4.73 | | 2.08 | | 1.93 | |
| $C_2H_4$ | 4.33 | | 6.47 | | 6.20 | |
| $C_2H_6$ | 1.14 | | 3.22 | | 3.60 | |
| $C_3H_6$ | 11.10 | | 21.39 | | 23.23 | |
| $C_3H_8$ | 34.24 | | 27.27 | | 21.33 | |
| $C_4H_{10}$ | 19.66 | | 14.98 | | 12.98 | |
| $C_4H_8$ | - | | 5.95 | | 10.78 | |
| $H_2$ | 0.02 | | 0.02 | | 0.02 | |
| $C_5H_{12}$ | 1.18 | | 0.90 | | 0.72 | |
| $C_5H_{10}$ | 0.14 | | 1.40 | | 2.79 | |

**Table S4.** Thermo-catalytic pyrolysis of PE in presence of HM with weight ratio (1:2) at different temperatures

| Temperature ($^0C$) | 450 | | 500 | |
|---|---|---|---|---|
| | Gas (%) | Solid (%) | Gas (%) | Solid (%) |
| Product gases | 87.77 | 12.23 | 85.36 | 14.64 |
| $CH_4$ | 0.64 | | 0.86 | |
| $CO_2$ | 1.96 | | 0.65 | |
| $C_2H_4$ | 4.42 | | 6.05 | |
| $C_2H_6$ | 4.39 | | 5.24 | |
| $C_3H_6$ | 6.12 | | 14.70 | |
| $C_3H_8$ | 51.42 | | 37.20 | |
| $C_4H_{10}$ | 16.28 | | 13.90 | |
| $C_4H_8$ | 1.45 | | 4.89 | |
| $H_2$ | 0.02 | | 0.04 | |
| $C_5H_{12}$ | 0.74 | | 0.70 | |
| $C_5H_{10}$ | 0.32 | | 1.12 | |

**Table S5.** Thermo-catalytic pyrolysis of PE in presence of Fe-HM with weight ratio (1:1) at different temperatures

| Temperature ($^0C$) | 400 | | 450 | | 500 | |
|---|---|---|---|---|---|---|
| | Gas (%) | Solid (%) | Gas (%) | Solid (%) | Gas (%) | Solid (%) |
| Product gases | 83.14 | 16.86 | 90.31 | 9.69 | 95.81 | 4.19 |
| $CH_4$ | 0.29 | | 0.64 | | 0.75 | |
| $CO_2$ | 9.99 | | 2.38 | | 1.69 | |
| $C_2H_4$ | 5.89 | | 5.98 | | 6.58 | |
| $C_2H_6$ | 1.95 | | 4.32 | | 4.47 | |
| $C_3H_6$ | 9.30 | | 24.89 | | 31.79 | |
| $C_3H_8$ | 39.39 | | 28.65 | | 20.15 | |
| $C_4H_{10}$ | 15.42 | | 13.79 | | 12.61 | |
| $C_4H_8$ | - | | 7.03 | | 13.17 | |
| $H_2$ | 0.16 | | 0.10 | | 0.11 | |
| $C_5H_{12}$ | 0.45 | | 0.74 | | 0.57 | |
| $C_5H_{10}$ | 0.31 | | 1.78 | | 3.93 | |

**Table S6.** Thermo-catalytic pyrolysis of PE in presence of Fe-HM with weight ratio (1:2) at different temperatures

| Temperature ($^0C$) | 450 | | 500 | |
|---|---|---|---|---|
| | Gas (%) | Solid (%) | Gas (%) | Solid (%) |
| Product gases | 90.74 | 9.26 | 86.54 | 13.46 |
| $CH_4$ | 0.89 | | 1.09 | |
| $CO_2$ | 2.02 | | 0.93 | |
| $C_2H_4$ | 4.09 | | 5.79 | |
| $C_2H_6$ | 5.17 | | 5.64 | |
| $C_3H_6$ | 13.52 | | 19.14 | |
| $C_3H_8$ | 45.26 | | 32.99 | |
| $C_4H_{10}$ | 13.45 | | 10.69 | |
| $C_4H_8$ | 4.74 | | 7.75 | |
| $H_2$ | 0.14 | | 0.12 | |
| $C_5H_{12}$ | 0.71 | | 0.53 | |
| $C_5H_{10}$ | 0.77 | | 1.88 | |

**Table S7.** Thermo-catalytic pyrolysis of PE in presence of Ru-HM with weight ratio (1:1) at different temperatures

| Temperature ($^0C$) | 400 | | 450 | | 500 | |
|---|---|---|---|---|---|---|
| | Gas (%) | Solid (%) | Gas (%) | Solid (%) | Gas (%) | Solid (%) |
| Product gases | 88.04 | 11.96 | 90.29 | 9.71 | 96.79 | 3.21 |
| $CH_4$ | 0.26 | | 0.64 | | 0.86 | |
| $CO_2$ | 7.37 | | 1.45 | | 5.25 | |
| $C_2H_4$ | 2.90 | | 6.78 | | 10.20 | |
| $C_2H_6$ | 1.66 | | 4.43 | | 4.78 | |
| $C_3H_6$ | 6.90 | | 25.14 | | 34.70 | |
| $C_3H_8$ | 54.74 | | 27.51 | | 11.71 | |
| $C_4H_{10}$ | 13.37 | | 12.81 | | 10.61 | |
| $C_4H_8$ | - | | 8.75 | | 13.13 | |
| $H_2$ | 0.11 | | 0.07 | | 0.09 | |
| $C_5H_{12}$ | 0.58 | | 0.60 | | 1.60 | |
| $C_5H_{10}$ | 0.15 | | 2.11 | | 3.85 | |

**Table S8.** Thermo-catalytic pyrolysis of PE in presence of Ru-HM with weight ratio (1:2) at different temperatures

| Temperature ($^0C$) | 450 | | 500 | |
|---|---|---|---|---|
| | Gas (%) | Solid (%) | Gas (%) | Solid (%) |
| Product gases | 89.9 | 10.1 | 90.18 | 9.82 |
| $CH_4$ | 1.36 | | 1.29 | |
| $CO_2$ | 2.35 | | 1.34 | |
| $C_2H_4$ | 3.54 | | 5.77 | |
| $C_2H_6$ | 7.19 | | 7.45 | |
| $C_3H_6$ | 7.06 | | 15.39 | |
| $C_3H_8$ | 56.24 | | 41.14 | |
| $C_4H_{10}$ | 9.46 | | 10.71 | |
| $C_4H_8$ | 1.84 | | 5.32 | |
| $H_2$ | 0.10 | | 0.08 | |
| $C_5H_{12}$ | 0.40 | | 0.52 | |
| $C_5H_{10}$ | 0.37 | | 1.16 | |

## DFT Studies

The Ru (0001) facet is chosen as it is the most stable and active facet of Ru metal centers. [1-3] The METE package was deployed previously and used in this work. [4] The dependent DFT calculations were carried out using the plane-wave based Vienna ab-initio Simulation Package (VASP) [5, 6] for energy minimization and structural optimization. The projector augmented-wave (PAW) method [7,8] was used to describe electron-ion interactions. Perdew-Burke-Ernzerhoff (PBE) exchange-correlation functional with the generalized gradient approximation (GGA) [8] was selected to evaluate energies. The D3 correction [9] was included for dispersion interactions. The cutoff energy of the plane-wave basis was set at 400 eV. The Gaussian smearing method with a smearing factor of 0.1 eV extrapolated the energies to 0 K. The Monkhorst-Pack scheme was followed by [10] with a 3×3×1 k-point mesh to sample the Brillouin zone. The electronic convergence for the self-consistent minimization was set at $10^{-6}$ eV, while the force convergence for ionic relaxation was set at 0.03 eV/Å.

### Entries for Reaction Mechanism

We examined all relevant species, including dehydrogenation and cracking steps, to gain insights into the most dominant reaction mechanism. Our search criteria for the species along the path is through its formation enthalpy- the lower the formation enthalpy is, the more stable the intermediate would exist on the surface and govern the overall reactions. **Table S9** lists all the considered intermediates in various steps. Intermediates in bold font are identified via our top-bottom search and labeled as the most stable ones and key to the reaction mechanism. Therefore, only these intermediates are included in **Figures 16** and **17** in the manuscript for clarity.

**Table S9. Probed reactions with calculated formation enthalpies**

| Adsorbate | Formation Enthalpy (kcal/mol) |
|---|---|
| $CH_3CH_2CH_2CH_3$* | -55.84 |
| **$CH_2CH_2CH_2CH_3$*+H*** | -61.11 |
| $CH_2CH_2CHCH_3$*+H* | -59.42 |
| $CH_2CH_2CH_2CH_2$*+H* | -58.06 |
| $CH_3CHCH_2CH_3$*+H* | -58.43 |
| **$CH_2CHCH_2CH_3$*+2H*** | -73.46 |
| **$CHCH_2CH_2CH_3$*+2H*** | -73.46 |
| **$CHCHCH_2CH_3$*+3H*** | -81.57 |

| | |
|---|---|
| $CH_2CCH_2CH_3$*+3H* | -79.28 |
| $CH_2CHCHCH_3$*+3H* | -80.05 |
| $CH_2CHCH_2CH_2$*+3H* | -80.27 |
| $CCHCH_2CH_3$*+4H* | -90.9 |
| **$CHCCH_2CH_3$*+4H*** | -96.06 |
| $CHCHCHCH_3$*+4H* | -93.08 |
| $CHCHCH_2CH_2$*+4H* | -86.95 |
| **$CHCH_2CHCH_3$*+3H*** | -81.57 |
| $CH_2CHCHCH_3$*+3H* | -80.05 |
| $CH_2CH_2CCH_3$*+3H* | -79.28 |
| $CH_2CH_2CHCH_2$*+3H* | -80.27 |
| $CCH_2CHCH_3$*+4H* | -90.9 |
| $CHCHCHCH_3$*+4H* | -93.08 |
| **$CHCH_2CCH_3$*+4H*** | -96.06 |
| $CHCH_2CHCH_2$*+4H* | -86.95 |
| CH*+$CHCH_2CH_3$* | -87.38 |
| CH*+$CCH_2CH_3$* | -100.62 |
| $CHCH_2$*+$CHCH_3$* | -97.00 |
| $CHCH_2$*+$CCH_3$* | -98.07 |

**Calculations using the Energy Span Model**

In the Energy Span Model (ESM), the Turnover Frequency is defined by an Eyring-style equation. This model simplifies a complex catalytic cycle by focusing on the "energetic span" ($\delta E$), which acts as the global activation barrier for the entire cycle.

The original equation used to define the TOF within this model is:

$$TOF = \frac{k_B T}{h} \exp\left(-\frac{\delta E}{RT}\right)$$

Where:

- $\delta E$ : the Energy Span
- $\frac{k_B T}{h}$: the frequency factor (prefactor).
- $R$ : the gas constant.
- $T$ : the absolute temperature in Kelvin.

1. Energy span model for mechanism of cracking at the terminal position on the Ru (0001) surface shown in **Figure 16**.

We have,

$$RT = (1.9872 \times 10^{-3\ \mathrm{kcal/mol}} \cdot \mathrm{K}) \times 773.15\ \mathrm{K}\}$$
$$RT \approx 1.5364\ \mathrm{kcal/mol}$$

Substitute the values into the exponent:

$$-\frac{\delta E}{RT} = -\frac{29.22}{1.5364} \approx -19.0184$$

$$e^{-19.0184} \approx 5.5006 \times 10^{-9}$$

Apply the Frequency Factor

Using the assumed prefactor of $1 \times 10^{9\ \mathrm{s}^{-1}}$:

$$TOF = (1 \times 10^{9\ \mathrm{s}^{-1}}) \times (5.5006 \times 10^{-9})$$
$$TOF = 5.5006\ \mathrm{s}^{-1}$$

2. Energy span model for mechanism of cracking at the middle position on the Ru (0001) surface is shown in **Figure 17**.

Similarly, we adapt the model as the following,

$$RT = (1.9872 \times 10^{-3\ \mathrm{kcal/mol}} \cdot \mathrm{K}) \times 773.15\ \mathrm{K}\}$$
$$RT \approx 1.5364\ \mathrm{kcal/mol}$$

$$\frac{\delta E}{RT} = \frac{38.13\ \mathrm{kcal/mol}}{1.5364\ \mathrm{kcal/mol}} \approx 24.8176$$
$$\exp(-24.8176) \approx 1.6666 \times 10^{-11}$$

$$TOF = (1 \times 10^{9\ \mathrm{s}^{-1}}) \times (1.6666 \times 10^{-11})$$
$$TOF \approx 0.01667\ \mathrm{s}^{-1}$$

**References**

1. Logadóttir, Á. and Nørskov, J.K. Ammonia synthesis over a Ru (0001) surface studied by density functional calculations. *Journal of Catalysis*, 2003,*220*(2), pp.273-279. DOI: 10.1016/S0021-9517(03)00156-8
2. Ciobica, I.M., Frechard, F., van Santen, R.A., Kleyn, A.W. and Hafner, J. A DFT study of transition states for C− H activation on the Ru (0001) surface. *The Journal of Physical Chemistry B*, 2000,*104*(14), pp.3364-3369. DOI: 10.1021/jp993314l
3. Nanba, Y., Ishimoto, T. and Koyama, M. Structural stability of ruthenium nanoparticles: a density functional theory study. *The Journal of Physical Chemistry C*, 2017,*121*(49), pp.27445-27452. DOI: 10.1021/acs.jpcc.7b08672
4. Xie, T., Wittreich, G.R., Curnan, M.T., Gu, G.H., Seals, K.N. and Tolbert, J.S.. Machine-Learning-Enabled Thermochemistry Estimator. *Journal of Chemical Information and Modeling*, 2024, *65*(1), pp.214-222. DOI: 10.1021/acs.jcim.4c00989
5. Kresse, G.; Furthmüller, J. Efficiency of ab-initio total energy calculations for metals and semiconductors using a plane-wave basis set. *Comput. Mater. Sci.* **1996**, *6* (1), 15-50. DOI: 10.1016/0927-0256(96)00008-0.
6. Kresse, G.; Joubert, D. From ultrasoft pseudopotentials to the projector augmented-wave method. *Phys. Rev. B* **1999**, *59* (3), 1758-1775. DOI: 10.1103/PhysRevB.59.1758.
7. Kresse, G.; Hafner, J. Norm-conserving and ultrasoft pseudopotentials for first-row and transition elements. *J. Phys.: Condens.Matter* **1994**, *6* (40), 8245. DOI: 10.1088/0953-8984/6/40/015.
8. Perdew, J. P.; Burke, K.; Ernzerhof, M. Generalized Gradient Approximation Made Simple. *Phys. Rev. Lett.* **1996**, *77* (18), 3865-3868. DOI: 10.1103/PhysRevLett.77.3865.
9. Grimme, S.; Ehrlich, S.; Goerigk, L. Effect of the damping function in dispersion corrected density functional theory. *J. Comput. Chem.* **2011**, *32* (7), 1456-1465. DOI: 10.1002/jcc.21759.
10. Monkhorst, H. J.; Pack, J. D. Special points for Brillouin-zone integrations. *Phys. Rev. B* **1976**, *13* (12), 5188-5192. DOI: 10.1103/PhysRevB.13.5188.
